# MNase titration reveals differences between nucleosome occupancy and chromatin accessibility

**DOI:** 10.1038/ncomms11485

**Published:** 2016-05-06

**Authors:** Jakub Mieczkowski, April Cook, Sarah K. Bowman, Britta Mueller, Burak H. Alver, Sharmistha Kundu, Aimee M. Deaton, Jennifer A. Urban, Erica Larschan, Peter J. Park, Robert E. Kingston, Michael Y. Tolstorukov

**Affiliations:** 1 Department of Molecular Biology, Massachusetts General Hospital and Harvard Medical School, Boston, Massachusetts 02114, USA; 2 Department of Biomedical Informatics, Harvard Medical School, Boston, Massachusetts 02115, USA; 3 Department of Molecular Biology, Cell Biology and Biochemistry, Brown University, Providence, Rhode Island 02912, USA

## Abstract

Chromatin accessibility plays a fundamental role in gene regulation. Nucleosome placement, usually measured by quantifying protection of DNA from enzymatic digestion, can regulate accessibility. We introduce a metric that uses micrococcal nuclease (MNase) digestion in a novel manner to measure chromatin accessibility by combining information from several digests of increasing depths. This metric, MACC (MNase accessibility), quantifies the inherent heterogeneity of nucleosome accessibility in which some nucleosomes are seen preferentially at high MNase and some at low MNase. MACC interrogates each genomic locus, measuring both nucleosome location and accessibility in the same assay. MACC can be performed either with or without a histone immunoprecipitation step, and thereby compares histone and non-histone protection. We find that changes in accessibility at enhancers, promoters and other regulatory regions do not correlate with changes in nucleosome occupancy. Moreover, high nucleosome occupancy does not necessarily preclude high accessibility, which reveals novel principles of chromatin regulation.

## Abstract (web summary)

Nucleosome positioning and chromatin accessibility are important contributors to the regulation of gene expression. Here the authors describe a method that allows the simultaneous measurement of nucleosome occupancy and chromatin accessibility in the same assay, revealing new features of chromatin organization linked to gene regulation.

## Graphical abstract

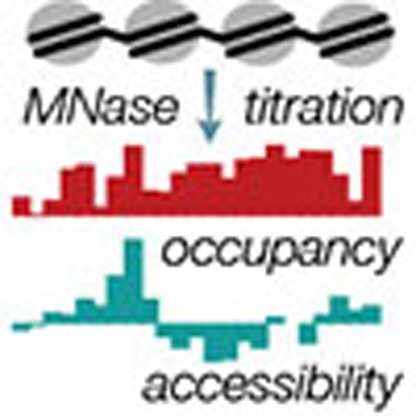

Nuclear factors facilitate the compaction of genomic DNA into chromatin^1^. DNA accessibility in chromatin is frequently controlled by nucleosomes, the basic repeating unit that contains about 150-bp of DNA and eight histone proteins^2^,^3^,^4^,^5^. The physical properties of nucleosomes vary throughout the genome. There are several families of ATP-dependent nucleosome remodelling complexes that can alter nucleosome stability, conformation and composition^6^. Histones are subject to covalent modifications and replacement with variants. This, along with other factors such as nucleosomal DNA sequence, can affect nucleosome stability and ability to form higher order structures^7^,^8^,^9^,^10^,^11^,^12^. Thus, nucleosomes are complex regulators of DNA accessibility, and mapping their genomic location and physical properties is of great importance for understanding the epigenome of a cell.

Current approaches for mapping nucleosome occupancy often rely on digestion with nucleases, most commonly micrococcal nuclease (MNase); however digestion assays are prone to technical issues that can hinder interpretation and sample-to-sample comparison of the results^13^,^14^. MNase displays sequence preference and its output is sensitive to even minor variation in enzyme activity^15^,^16^,^17^. This can result in apparent differences in nucleosome occupancy that are not dependent upon biological regulation but upon technical differences in the digestion^18^,^19^. Experimental and computational approaches have been proposed to deal with these digestion biases. These approaches attempt to ‘standardize' the occupancy maps through optimizing digestion conditions, normalizing the generated data or using different reagents to fragment chromatin^20^,^21^,^22^,^23^,^24^.

We have developed a new methodology to quantify chromatin accessibility across the genomes of various complexity by utilizing different MNase concentrations. Differences in nucleosome occupancy measured under light and deep-digestion conditions have been used previously to map ‘fragile' nucleosomes, that is, nucleosomes seen well only under light-digestion conditions, in distinct regulatory regions in yeast^19^,^25^. These studies demonstrated that using as few as two independent MNase concentrations can produce biologically relevant information that is not apparent with one MNase condition alone. We expand upon this concept to generate a novel approach that uses experimental and computational methods for detailed analysis of the physical organization of chromatin. This approach uses a range of MNase concentrations to create independent data sets and considers these data sets in relation to each other. This method does not rely on nucleosome occupancy as a stand-alone metric, quantifying DNA accessibility instead. It leverages the dependence of the measured nucleosome signal on the level of digestion by MNase, rather than attempting to alleviate its impact. When combined with histone chromatin immunoprecipitation (ChIP), this method allows comparison of the sites bound by nucleosomes to those bound by other regulatory components. We show that this metric offers substantially more information than can be gained solely by measuring nucleosome occupancy.

## Results

### Data generation

**Figure 1:**
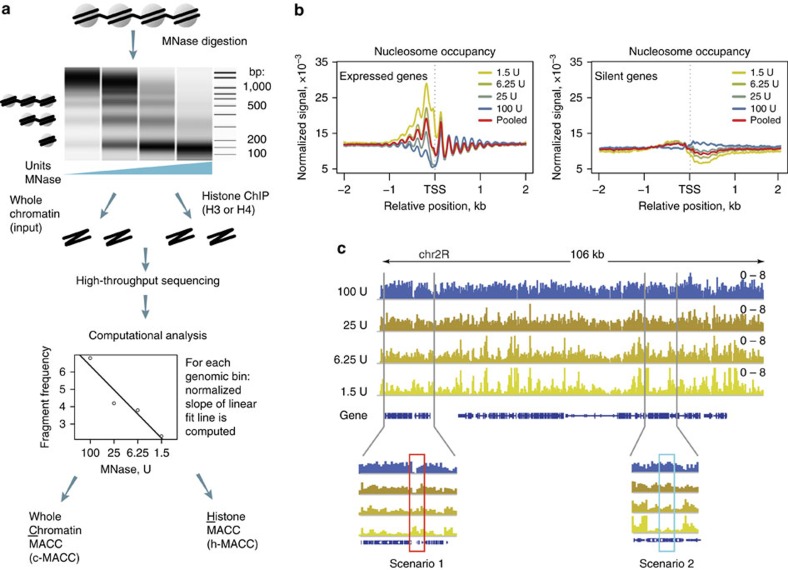
MNase concentration affects the results of nucleosome occupancy profiling. (**a**) The workflow of the MNase accessibility (MACC) assay. Upper panel: capillary electrophoresis of digestion products from a typical MNase titration experiment. Middle: the digestion fragments are separated into two samples, those sequenced as whole-chromatin extract, and those sequenced after additional enrichment for histone-associated DNA using chromatin immunoprecipitation. Lower panel: quantification of the chromatin response measured in a four point MNase titration at a single location in the genome. Linear regression is fitted to the fragment frequencies obtained for a 300-bp bin at each MNase titration point. The logarithmic scale of MNase concentrations was used to obtain equidistant distribution of experimental points. The regression slope is used as a measure of DNA accessibility for MNase at this locus. Additional data-correction step was used to normalize MACC for GC content of underlying DNA sequence to address possible bias due to MNase sequence preferences (see Supplementary Information for detail). (**b**) MNase-seq profiles around TSS (transcription start sites) for expressed (left) and silent (right) genes. Yellow-blue colour scheme indicates MNase concentration levels (1.5, 6.25, 25 and 100 U), with bright yellow corresponding to the lowest concentration and dark blue corresponding to the highest. The red line depicts an averaged profile. (**c**) An example locus showing frequency profiles of the digestion fragments obtained from MNase titration. The colour scheme is the same as in **b**. Gene structure is indicated at the bottom of the plot. Two regions are expanded to illustrate the major scenarios of the chromatin response to MNase titration, with scenario 1 (‘open', red box) showing increasing nucleosome signal with decreasing MNase levels, and scenario 2 (‘closed', blue box) showing decreasing nucleosome signal with decreasing MNase levels.

To obtain a comprehensive map of DNA accessibility, we digested chromatin from *Drosophila melanogaster* S2 cells using four different concentrations of micrococcal nuclease (MNase titrations). An exponential titration series of MNase was used (1.5, 6.25, 25 and 100 U) that was chosen to generate a small amount of mononucleosomal fragments at the lowest MNase concentration and predominantly mononucleosomal DNA at the highest concentration of MNase (Fig. 1a, upper panel). Following a low-stringency size selection that removed most DNA over 1,000 bp (see Methods section), the remaining digestion products were subjected to library construction and paired-end sequencing. The total tag counts were at least 25 million paired-end reads per digestion point (Supplementary Table 1), with an average fragment length (∼150 bp, Supplementary Fig. 1) that showed some dependence upon degree of digestion but was consistent with mononucleosomal DNA.

Previous studies have shown that 150 bp fragments generated by MNase digestion normally are caused by protection by mononucleosomes, but also can be caused by protection by other chromosomal proteins. To enrich the pools in nucleosomal DNA we developed a second protocol that includes histone immunoprecipitation before sequencing (Fig. 1a).

These data can be analysed by counting the fragments that map to each genomic location and, upon normalization for total library size, these counts are commonly interpreted as occupancy values characterizing genomic locations relative to each other. The data from the titration points can be used to determine how that relative occupancy changes at distinct MNase concentrations. Below we demonstrate that, as has been observed previously, occupancy changes in a complex manner at distinct MNase concentrations. This makes it difficult to make reproducible measurements of occupancy in different experiments, as MNase activity varies significantly with experimental conditions. Instead we focus on how occupancy changes with MNase titration in individual regions of the genome to assign a parameter that measures that behaviour (Fig. 1a, lower panel; also see below). We term this parameter MACC (MNase accessibility), and calculate MACC using chromatin as input (called ‘c-MACC') or histone immunoprecipitates as input (‘h-MACC'). This approach leverages MNase digestion variability by focusing on how digestion changes, and hence how DNA accessibility changes, rather than measuring nucleosome occupancy *per se*. We demonstrate that c-MACC and h-MACC give similar results over most of the genome, that MACC measures a separate characteristic than occupancy, and that comparing c-MACC and h-MACC offers insight into regulation.

### Two scenarios of chromatin response to MNase titration

We determined the distribution of nucleosome occupancy around transcription start sites (TSS) as an initial test of MNase titration. Previous studies have shown a nucleosome ‘free' region at the TSS surrounded by nucleosomes at −1 and +1 positions, and defined arrays of nucleosomes propagating into the gene. We found (Fig. 1b, c-MACC; similar results for h-MACC are shown in Supplementary Fig. 2) that these features depend on the MNase concentration used. For instance, the measured occupancy of the ‘−1' nucleosome immediately upstream of the TSS is highest under light-digestion conditions^17^, while nucleosome occupancy inside genes is highest under deep-digestion conditions. Averaging the data from all titrations (‘pooled' line, Fig. 1b) produces a shape similar to those reported previously^26^,^27^. Similarly, measurements of occupancy change across the whole genome with MNase titration (Fig. 1c), with some regions showing increased occupancy at low MNase (scenario 1) and other showing increased occupancy at high MNase (scenario 2).

These data demonstrate two opposing scenarios of chromatin response to MNase titration: the number of sequenced DNA fragments from a given locus might increase or decrease with increasing MNase concentration. For example, under light-digestion conditions, regions with accessible DNA are preferentially released by MNase (such as the ‘−1' nucleosome); however these regions get digested to fragments of sub-nucleosomal sizes (which are excluded in our library construction protocol) under deeper digestion conditions (scenario 1) (Supplementary Fig. 1). In contrast, regions of low DNA accessibility (such as transcribed regions) are not released by MNase under light-digestion conditions. High MNase activity is required to digest these loci into mononucleosomal fragments (scenario 2).

To measure the response of a genomic locus to MNase titration, we calculate a metric that reflects the rate of the change in signal at a particular locus in response to decreasing MNase concentration (Fig. 1a, lower panel). This metric, MACC, represents the slope of the linear regression fitted to the digestion fragment frequencies obtained for each MNase concentration at any one region (bin) in the genome. The slope can have both positive and negative values and we expect MACC positive values to correspond to scenario 1 described above (accessible chromatin) and MACC negative values to correspond to the scenario 2 (inaccessible chromatin). This quantitative metric characterizes the response of a specific chromatin locus to MNase probing and can be interpreted as a measure of the ease with which a factor of the size of MNase can access the genomic DNA.

### A novel metric to quantify chromatin accessibility

**Figure 2:**
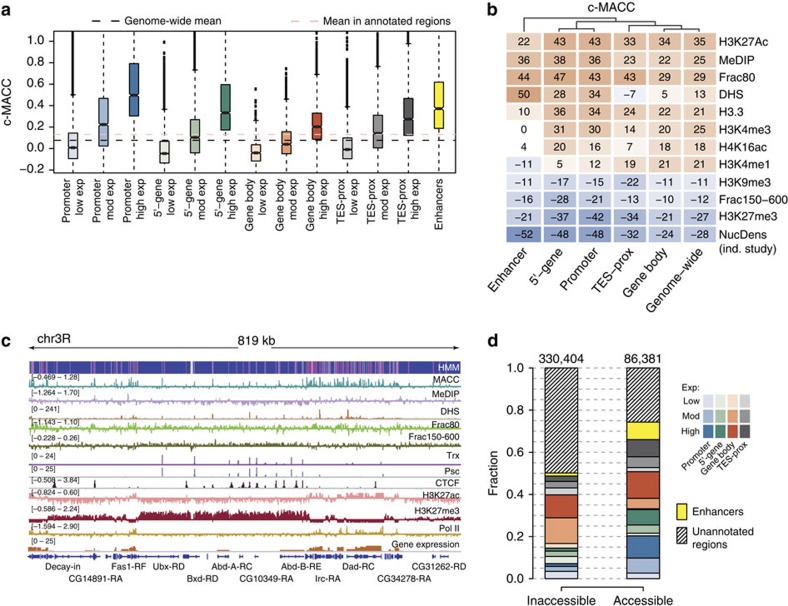
Relation of MACC to other markers of chromatin structure and annotated regions of the genome. (**a**) Distribution of MACC values within annotated regions. The results are shown for promoters (1 kb upstream of TSS, blue), 5′-ends of genes (1 kb downstream of TSS, green), gene bodies (red), regions around transcription end sites (±1 kb, grey), and enhancers (identified by modENCODE consortium for S2 cells^27^, yellow). The shade of the colour within each group of regions indicates the magnitude of expression level. For overlapping regions the category was selected using the following priority rule: enhancer>promoters>5′-gene>TES-prox>gene bodies. (**b**) A heatmap depicting relation between MACC and chromatin markers computed genome wide and within annotated regions. The data on histone marks and DNaseI hypersensitivity (DHS) were taken from Kharchenko *et al*.^45^. The data on salt fractionation of chromatin, H3.3 and nucleosome density (NucDens) were taken from Henikoff *et al*.^8^. Also, the chromatin accessibility accessed using an independent method^29^ was used for comparison (MeDIP). The values appearing in the heatmap cells represent Pearson's correlation coefficients multiplied by 100. Colour scale encodes the same values, with red and blue colours standing for positive and negative correlations, respectively. (**c**) Profiles of MACC, histone marks, chromatin-modifying proteins and physical properties of chromatin at a ∼800-kb locus on chromosome 3R of the fly genome. The blue and magenta track at the top of the snapshot shows assignment of the 2-state Hidden Markov Model (HMM) generated using the MACC profile. The magenta and blue colours correspond to the accessible and inaccessible states, respectively. (**d**) Distribution of MACC states in genomic regions. Accessible and inaccessible states were identified with HMM for 300-bp bins. Stacked bars represent fractions of the bins assigned to each state in the corresponding regions, defined as in **a**. The numbers of bins in each state are shown above the bars.

We hypothesized that MACC scores might relate to gene regulation, and therefore determined how MACC scores are distributed in regulatory regions of the genome (see Fig. 2a, Supplementary Fig. 3b for c-MACC and Supplementary Figs 2, 3a for h-MACC values). On average, the highest MACC values (high MNase accessibility) were detected in regions associated with active transcription, specifically gene promoters and active enhancers. Gene bodies also show higher accessibility when transcribed, but this dependence was less pronounced than it was in promoters.

We explored the relation of MACC scores to chromatin markers associated with regulation (see Fig. 2b for c-MACC and Supplementary Fig. 4 for h-MACC). MACC positively correlated with indicators of active chromatin (for example, histone marks H3K4me3 and H3K27ac or the low-salt fraction of chromatin characteristic for unstable nucleosomes). Reciprocally MACC negatively correlated with indicators of silent chromatin (for example, H3K27me3 and H3K9me3). The magnitude of such correlations was increased at the sites of pronounced MACC peaks and dips, respectively (Supplementary Fig. 4), further supporting the association of positive and negative MACC values with the corresponding markers.

While the relationship of MACC to most chromatin metrics is consistent across regions and is also consistent with expectation based upon known regulation, the relationship of MACC to DNase hypersensitivity (DHS) is complicated. The correlation between MACC and DHS reaches maximal positive values at enhancers, indicating that both metrics are suitable for identification of these regions, and reaches lowest values at the 3′-ends of the genes (Fig. 2b). Thus, MACC and DHS measurements offer distinct insights into accessibility, presumably reflecting the distinct nature of the different nucleases used in the two methodologies.

The relation between MACC and other chromatin features is further exemplified in Fig. 2c. At the scale of hundreds of kilobases, the MACC profile corresponds closely to the domains of H3K27me3 and H3K27ac enrichment, marking ‘transcriptionally silent' and ‘transcriptionally active' chromatin, respectively. Formation of such domains means that loci with similar MACC values tend to be located close to each other, often forming continuous stretches. To investigate this effect further, we used a machine-learning approach based on the construction of a Hidden Markov Model (HMM) with two states (that is, generating binary annotation of the genome): ‘accessible', mostly positive values of MACC in a region, and ‘inaccessible', mostly negative values of MACC (see HMM track in Fig. 2c). We estimated that the accessible state covers about 21% of the genome and, in line with our previous findings, is preferentially associated with active chromatin (Fig. 2d; Supplementary Fig. 4). We note that the stretches of the accessible and inaccessible states are longer than expected by chance (Supplementary Fig. 4), suggesting a possible regulatory role for such clustering.

We conclude that MACC provides both a quantitative and qualitative description of chromatin accessibility and that it captures the expected trends for ‘active' and ‘repressed' regions of the genome. It provides additional information by assigning an accessibility metric to all parts of the genome.

### MACC characterizes histone and non-histone protein binding

Protection from MNase digestion can result both from histones in nucleosomes and from non-histone protein (NHP) factors. Thus, c-MACC scores are expected to reflect a combination of these types of protection. Consistent with this expectation^28^,^29^, chromatin-bound NHPs frequently occupy sites of locally increased accessibility (peaks) even if these proteins are associated with gene silencing (for example, see binding peaks of Polycomb protein Psc in Fig. 2c; the average c-MACC profiles around binding sites of selected proteins are shown in Supplementary Fig. 5). We therefore determined how h-MACC measurements, which include a histone immunoprecipitation step to measure MNase accessibility to nucleosomal sites, compares with c-MACC, which measures accessibility at all sites in the genome. For validation purposes, the h-MACC profiles for histones H3 and H4 were produced; these profiles were strongly correlated (Supplementary Fig. 6) and hence, for brevity, we include only the H3 results.

**Figure 3:**
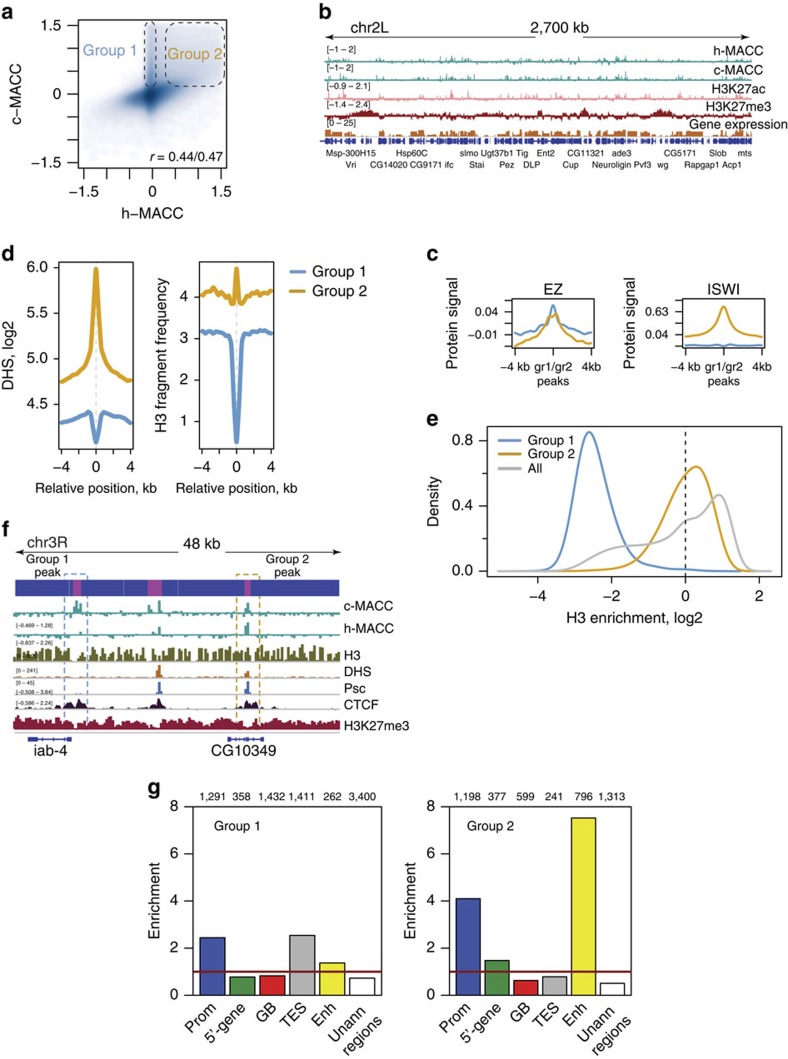
Chromatin accessibility is modulated through DNA protection by histone and non-histone factors. (**a**,**b**) Comparison of h-MACC (based on histone enrichment data) and c-MACC (based on whole-chromatin digestion data). (**a**) Scatterplot showing h- and c-MACC values in all analysed bins. Ovals indicate the sets of genomic loci characterized by either high values of c-MACC and low values of h-MACC (group 1) or by high values of both h- and c-MACC (group 2). (**b**) A genome browser screenshot featuring a ∼2.5 Mb region from chromosome 2 L. (**c**) Overlap of the MACC peaks from group 1 or group 2 (blue and orange lines respectively) with protein binding. The results for two proteins are shown as examples (see Supplementary Figs 7–9 for a more comprehensive analysis). (**d**) Distributions of DHS and H3 signals around the MACC peaks from groups 1 or 2. (**e**) Distribution of the H3 enrichment levels at the sites associated with group 1 (blue), group 2 (orange) and all genomic bins (grey). The dashed vertical line provides reference of no ‘enrichment'. (**f**) An example of the protein binding reflected in the MACC profiles, featuring protein binding with (blue rectangle) and without (orange rectangle) nucleosome displacement. (**g**) Genomic distribution of the MACC peaks from groups 1 and 2. The enrichments were computed relative to the expected values for each type of genomic regions. See Methods for region definitions. The horizontal red lines provide reference of 1 (‘no enrichment').

Overall, h-MACC and c-MACC show a high degree of similarity (Fig. 3a,b; Supplementary Fig. 6). Given that the major results described above were observed using both h-MACC and c-MACC scores (Supplementary Fig. 4), these findings indicate that the majority of the DNA protection is primarily determined by histone–DNA interactions.

Deviation between h- and c-MACC values is mostly observed for sites with positive c-MACC values (Fig. 3a, upper part; also *cf.* c- and h-MACC distributions at TSS, Supplementary Fig. 3a,b). Specifically, a subset of sites (group 1, ∼3% of all bins) show increased c-MACC values while h-MACC is close to zero, implying a role for NHPs in generating these sites. We compared this subset to a subset of comparable size that is characterized by increased values of both c- and h-MACC (group 2). Given that a likely cause of the difference between c-MACC and h-MACC is binding by NHP factors, and that these components frequently bind in distinct peaks, we limited further analysis to the sets of peaks belonging to groups 1 and 2 (about 8,000 and 5,000 peaks, respectively).

To test the hypothesis that certain of these peaks are associated with NHP binding, we examined their overlap with known NHP-binding sites using publicly available data (see Methods section)^30^. We found that the MACC peaks from both groups overlapped with NHP-binding sites (as exemplified in Fig. 3c, Supplementary Fig. 7), with group 2 showing stronger enrichment in the proteins associated with active transcription (for example, RNA Pol II) and chromatin remodelling (for example, ISWI/NURF) (Fig. 3c; Supplementary Fig. 8). The precise fraction of MACC peaks overlapped by NHP-biding sites cannot be accurately estimated, as it is a function of the thresholds used in the analysis (Supplementary Fig. 9) and data sets are only available for a subset of NHPs; however, we conclude that such overlap is frequent in fly chromatin (for example, it reaches 28% and 77% for groups 1 and 2, respectively, when *Z*-score=3 is used to call protein binding). The available data sets focus on components of regulatory factors that are used broadly (for example, general transcription factors, chromatin modifying complexes, and so on) and are underrepresented in data on binding of gene-specific DNA-binding factors. This limits our ability to determine the extent to which MACC might reflect binding by gene-specific factors, which is anticipated from previous studies to play a key role in accessibility^30^.

To explore the relative contribution of histones and NHP binding to the sites in groups 1 and 2, we quantified H3 levels for these sites. As expected by their definition, loci from group 1 are almost uniformly depleted of H3, loci from group 2 are associated with a peak in the H3 profile (Fig. 3d,e; Supplementary Fig. 10). This suggests that generation of the MNase fragments in group 1 is caused by NHP binding. Group 1 sites are unlikely to reflect naked DNA, as these sites are depleted in DHS (Fig. 3d). The anti-correlation of group 1 sites and DHS sites offers further support to the notion that MACC and DHS measures distinct chromatin properties. The overall H3 enrichment at group 2 sites is about 40% lower than most frequently observed for nucleosome-occupied loci genome wide (Fig. 3e). This might reflect distinct cell populations, with some cells containing nucleosomes at these sites and others lacking nucleosomes at these sites. The enrichment of group 2 sites for binding by general factors, including chromatin modifying factors, indicates that some of these sites might be co-bound by nucleosomes and factors that function on nucleosomal DNA.

These sites exhibit distinct patterns of enrichment in relation to regulatory features (Fig. 3g; Supplementary Fig. 11): group 2 showed strongest enrichment at gene enhancers and promoters, group 1 was more enriched at promoters and transcription end sites (TES). Sites from both groups show preference for genes with higher expression (Supplementary Fig. 11).

We used a number of experimental and computational approaches to validate the group 1 and 2 sites. To this end we ruled out that these sites are artifacts of the experimental platform used in our study by performing MNase-qPCR experiment (Supplementary Fig. 12; see Methods section for further details on MACC validation). Analysis of the distributions of the fragment sizes at the group 1 and 2 sites showed that group 1 sites are associated with shorter fragments than genome in bulk at deep-digestion conditions, providing further support for the idea that these sites feature non-nucleosomal protection (Supplementary Fig. 13). Finally, we performed sequence analysis of these sites and found that while group 1 sites are less GC rich and group 2 sites are more GC rich than the genome on average the sequence bias in the MNase digestion does not considerably contribute to the presence of these sites in the chromatin (Supplementary Fig. 14).

A detailed analysis of the chromatin organization around sites from group 1 and group 2 revealed different patterns of nucleosome accessibility (h-MACC) for these groups (Supplementary Fig. 15). Accessible nucleosomes (positive h-MACC) flanking either a group 1 or a group 2 peak were frequent; however, this pattern was considerably more prevalent for group 2 (85% of group 2 peaks versus 31% of the group 1 peaks). The relative abundance of these patterns relates to NHP identity (Supplementary Fig. 15), for example group 2 sites surrounded by accessible nucleosomes are enriched with the remodeler NURF, transcription factor GAF, RNA Pol II and several histone-interacting proteins. Thus, information from c-MACC and h-MACC can be combined to form hypotheses for how regulatory factors might impact the characteristics of surrounding nucleosomes.

### h-MACC distinguishes nucleosome occupancy and accessibility

We determined how nucleosome occupancy relates to the accessibility metric provided by h-MACC. Nucleosome occupancy obtained in assays using one mononucleosome pool generated by MNase digestion differs from that obtained with distinct MNase concentration (see above). To measure nucleosome occupancy, we therefore used the averaged frequency of paired-end reads in 300-bp bins over all MNase titration points. This approximates the signal that could be obtained if the libraries corresponding to the individual titration points were pooled together to produce a single map of nucleosome occupancy. Thus, occupancy and h-MACC are measured using the same data.

**Figure 4:**
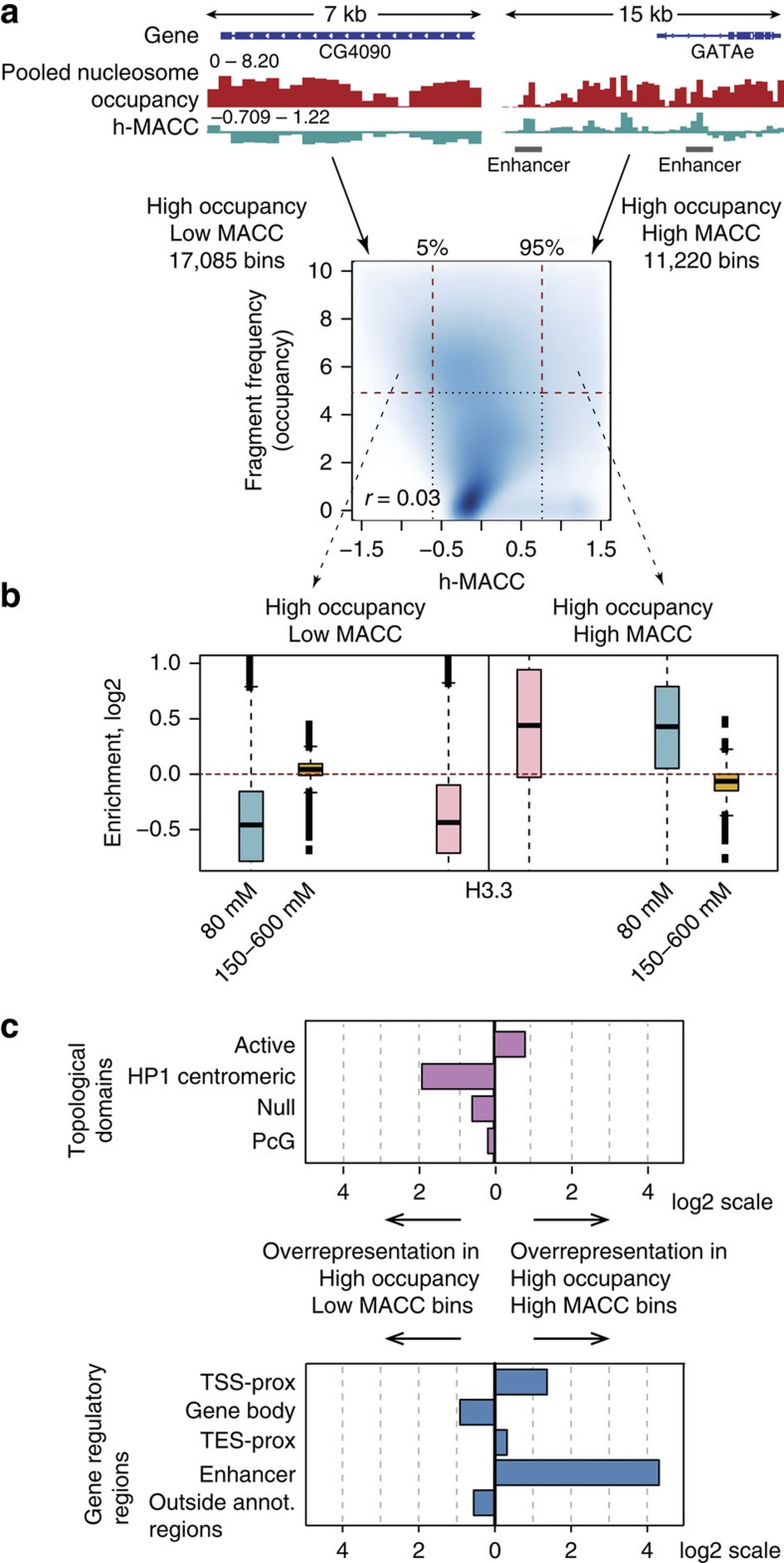
MACC is a better predictor of DNA accessibility than nucleosome occupancy. (**a**) Examples (upper panels) and scatterplot (lower panel) showing association between MACC and ‘pooled' nucleosome occupancy. A single point on the scatterplot corresponds to one 300-bp bin (all bins for which MACC was estimated were included in this analysis). The Pearson's correlation coefficient between MACC and nucleosome occupancy is shown at the bottom. The dashed horizontal line indicates the high nucleosome occupancy cutoff (top 20% of bins), and the vertical dashed lines indicate MACC value thresholds for top and bottom 5% of bins. The numbers of genomic bins in each region are indicated on the plot. (**b**) Enrichment of salt-extracted chromatin fractions and H3.3 histone variant in the high occupancy bins shown in **a**. The 80 mM ‘active nucleosome' fraction is blue, the 150–600 mM ‘stable' or ‘repressed' fraction is orange, and the H3.3 enrichment is pink. The boxplots on the left and right sides of the figure (separated by the vertical black line) correspond to the bins with low and high MACC values respectively. Enrichment is plotted on a log2 scale and red horizontal line is placed at 0. (**c**) Comparison of the enrichment of two selected sets of bins in topological domains (upper panel, purple) and annotated genomic regions (lower panel, blue): TSS proximal (±1 kb), gene body, TES-proximal (±1 kb), and enhancers. For each bin set we calculated the fraction of bins overlapping a given region and the ratio of these fractions is shown on the plot.

We tested the correlation between the ‘pooled' nucleosome occupancy and h-MACC over the genome (Fig. 4a). Surprisingly, we observed that genomic locations with high ‘pooled' occupancy values, which are traditionally thought of as ‘closed', could have both low and high levels of MACC. This suggests existence of a range of accessibility at regions previously thought to be inaccessible. Thus chromatin can be accessible to MNase even when it has high nucleosome occupancy.

One interpretation of this finding is that the nucleosomes at the genomic locations with both high accessibility and occupancy (right part of Fig. 4a) have different properties than nucleosomes in regions of low accessibility. To explore this hypothesis, we used the data produced by the salt fractionation approach, which separates nucleosomes of different stability^8^. We observed that sites with high nucleosome occupancy and high MACC values are enriched in the low-salt fraction, which represents less stable nucleosomes. The sites with high occupancy and low MACC values are enriched in the high-salt fraction, representing more stable nucleosomes (Fig. 4b). The detected difference in the nucleosome stability can be related to the differences in their composition, for example, presence of the histone variant H3.3 (Fig. 4b), which has been reported to be associated with less stable nucleosomes^8^. We also observed that the Drosophila topological domains^31^,^32^ comprising active chromatin are enriched in the accessible sites, while the domains of repressed chromatin are enriched in the inaccessible sites (Fig. 4c). This observation suggests that three-dimensional packaging of the genome can be another factor contributing to the difference in the chromatin accessibility at local level.

Regions that have high nucleosome occupancy and high accessibility significantly overlap with the group 2 sites associated with NHP binding (*P*<10^−15^, Fisher's exact test; Supplementary Fig. 16) and are distributed non-randomly in the genome, which suggests a role in regulation. Regulatory regions such as enhancers and promoters are enriched in the sites of high occupancy and high accessibility, while gene bodies and unannotated genomic regions are enriched in the sites of high occupancy and low accessibility (Fig. 4c). Thus, the information on DNA accessibility, obtained by considering titration points independently, allows a more detailed description of chromatin structure than that provided by the ‘pooled' nucleosome occupancy alone.

### Use of c-MACC to characterize mammalian genomes

The sequence composition and organization of genomes of different organisms vary considerably, prompting us to examine whether c-MACC provides useful information in mammalian cells. An important issue with using either c-MACC or h-MACC to characterize mammalian cells is cost, as bar-coding of samples, which can be used for MACC in Drosophila, cannot be done to the same extent in mammals due to depth of coverage issues. We chose to use c-MACC in several mammalian sources to determine proof-of-principle in mammals, as c-MACC offers a broader set of information than h-MACC. We generated and analysed MNase titration data for two separate mouse ES cell line, two distinct mouse neural progenitor lines, and human K562 cells, which have extensive data associated with them from ENCODE.

**Figure 5:**
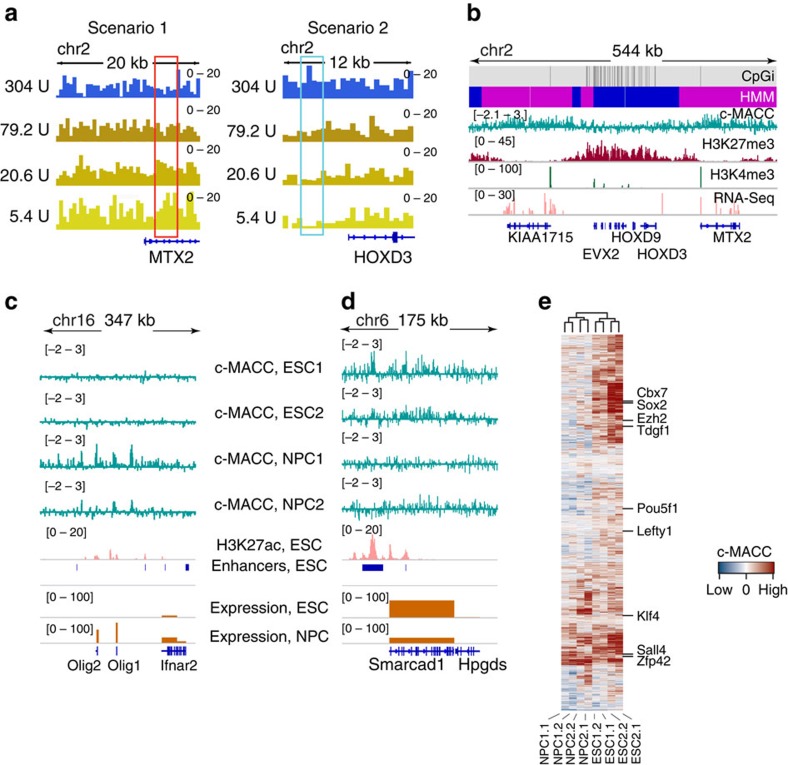
MACC profiling in the mammalian genomes. (**a**) A snapshot of digestion fragment frequency profiles obtained with four MNase concentrations. A colour scheme represents MNase concentration levels as in Fig. 1 (see legend of Fig. 1c for details). (**b**) Properties of chromatin around HOXD cluster in the human genome (∼540 Kb locus on chromosome 2). The grey and black track (CpGi) at the top of the plot indicates locations of CpG islands. The blue and magenta track (HMM) represents the distribution of inaccessible (blue) and accessible (magenta) states assigned by the 2-state HHM model based on the c-MACC profile (cyan). Other tracks show corresponding data as indicated on the plot. (**c**,**d**) MACC profiles ‘predict' active and silenced regions in mouse ESCs and NPCs. c-MACC (cyan) has increased levels in NPCs around genes Olig1 and Olig2, which are active in this cell type (**c**). An opposite pattern of c-MACC is observed around gene Smarcad1, which has higher expression in ESCs (**d**). c-MACC is also increased at the active enhancers characterized by the high levels of H3K27ac (pink). (**e**) Heatmap representing c-MACC values at ESC enhancers. c-MACC values computed for independent replicates were used for this analysis (marked with additional numbering at the samples names). Examples of the genes closest to the shown enhancers are given on the right. The analysed samples clustered according to their cell types (see dendrogram at the top).

As was found in the fly, the two major scenarios of response to MNase titration, increasing or decreasing signal with increasing MNase digestion (Fig. 1), also prevailed in the mammalian genomes (Fig. 5a; Supplementary Fig. 17). Similarly, c-MACC in mammalian genomes is elevated in regulatory regions, such as promoters and enhancers, and is positively correlated with transcription (Supplementary Fig. 17). This association is further supported by the comparison of the c-MACC profile with the profiles of several markers of ‘active' and ‘silent' chromatin (Fig. 5b–d). MACC is decreased across HOX loci in K562 cells, a known repressed region, consistent with compaction of these loci during repression^33^.

To determine whether c-MACC provides consistent results in distinct mammalian cell lines with similar characteristics, we performed c-MACC on two mouse ESC lines (ESC1 and ESC2), on neuronal precursor cells (NPCs) derived *in vitro* from ESCs (NPC1), and on NPCs isolated from mouse embryonic brain (NPC2). We found that regions of c-MACC were consistent between the two pluripotent lines and between the two NPC lines, but differed considerably between the pluripotent and differentiated lines. For example, genes active in NPCs but inactive in ESCs had higher c-MACC in NPCs (Fig. 5c) and vice versa (Fig. 5d). To determine the generality of this observation, we examined enhancers annotated in ESCs^34^ and performed unsupervised clustering of these enhancers by c-MACC score. Biological replicates of the both ESC lines clustered together, as did biological replicates of the two NPC lines (Fig. 5e). Most of the analysed ESC enhancers showed decreased c-MACC in NPCs. We conclude that c-MACC produces consistent results in mammalian cells, and that the basic observations are similar in mammals and flies. Thus, c-MACC is applicable to the analysis of chromatin structure in different organisms.

## Discussion

We introduce an integrative approach to investigate chromatin structure that uses measurements of sensitivity to MNase titration across the genome. Traditionally MNase assays have been used to profile nucleosome occupancy. Because apparent nucleosome occupancy varies with the level of digestion, comparing and interpreting individual experiments performed with a single MNase concentration is difficult to do with confidence. The level of chromatin digestion using MNase is sensitive to even slight variation in enzyme activity, and different nucleosomes are released by MNase cleavage with different efficiency. We present an approach to MNase digestion that is self-normalized and focused on DNA accessibility rather than on nucleosome occupancy. This approach is more robust to experimental variability and its outcome is easier to interpret.

The MACC protocol directly addresses the relationship between nucleosome occupancy and DNA accessibility since both of them are profiled in the same assay. We demonstrate that in addition to the expected scenario, where high occupancy corresponds to low DNA accessibility, regulatory loci often have chromatin arrangements characterized by relatively high nucleosome occupancy with high accessibility. We infer that this arrangement is facilitated by nucleosomes of low stability. These regions are often enriched in marks of active chromatin (for example, histone variants H3.3 and H2A.Z or modifications including H3K27ac and other acetylation events), which might contribute to high accessibility. Another factor contributing to high accessibility of such regions can be an increase in inter-nucleosomal spacing (linkers)^22^ due to chromatin remodelling^22^. These possibilities are not mutually exclusive, and while we observed only moderate dependence of the linker size on the digestion level (Supplementary Fig. 1), we note that MACC scores would reflect cumulative effect of both mechanisms.

MACC provides advantages over other methods for studying the physical state of chromatin. First, it allows profiling of the entire genome, rather than focusing on local regions of ‘open' chromatin (*cf.* DHS, H3.3, ATAC-Seq)^8^,^35^,^36^; thus, active enhancers and Polycomb-repressed regions can be identified in one experiment and quantitatively compared. Second, it allows description of DNA accessibility across continuous regions of the chromosome, thus allowing detection of up to mega-base domains that have similar characteristics. Finally, it is technically straightforward, since it is based on an enzymatic reaction and does not involve additional steps, such as introduction of a transgene into the cell (H3.3) or *in vitro* methylation of genomic DNA (MeDIP footprint)^29^.

An important point is that the cost associated with the MACC approach does not have to be much higher than the cost of the ‘traditional' nucleosome profiling performed by many labs. Indeed, the nucleosome occupancy estimated in the MACC assay is based on the pooled set of tags produced in all digests and, thus, requires the same number of tags as the occupancy in the case of ‘traditional' profiling. Individual digestion profiles are compared for calculation of MACC scores, however, these scores are calculated in the 100–300 bp bins and therefore this requires fewer tags than the evaluation of nucleosome occupancy at base-pair resolution. As an illustration, one can compare the number of tags used for MACC assessment in a mammalian genome in our study (∼200–250 millions of paired-end tags) with the number of tags used in the ATAC-Seq profiling of GM12878 cells (∼200 millions of paired-end tags)^36^ and with the number of tags used in the MNase-Seq study on the same cell type (∼4 billions of single-end tags)^37^.

A comparison of MACC profiles computed for the whole-chromatin digest and for the samples enriched in a core histone reveals sites where chromatin accessibility is affected by the factors other than nucleosomes. Analysis at such sites allowed detection of different modes of protein binding, with and without predominant nucleosome displacement. These modes have patterns that differ with regulation, suggesting that MACC profiling may have a wide range of applications in generating hypotheses for regulatory mechanism. For instance, binding of pioneer transcription factors to nucleosomal DNA during initiation of cell-fate changes and subsequent chromatin rearrangements may involve different modes of protein binding detectable by the MACC approach^30^. MACC is therefore a straightforward metric that can be used with or without histone immunoprecipitation to characterize features of the genome in ways that differ from previous methodologies.

## Methods

### MNase titration in Drosophila S2 cells

Drosophila S2 cells were grown in Schneider's medium supplemented with 10% heat-inactivated fetal bovine serum (FBS) at 28 °C. For fixation, 10^7^ S2 cells were pelleted and rinsed twice in phosphate-buffered saline (PBS). Cells were crosslinked in 1.1% formaldehyde in PBS for 10 min at room temperature, tumbling end over end in a volume of 10 ml. After addition of 50 μl of 2.5 M glycine, the cells were tumbled at room temperature for 2 min to quench the reaction. Cells were next rinsed twice with cold PBS before pelleting and flash freezing in liquid nitrogen. For MNase digestion, the cell pellet was resuspended in PBS with 0.1% Triton X-100 (PBS–TX). Digestion of 10^6^ cells per titration point took place in a volume of 400 μl PBS–TX supplemented with 1 mM CaCl_2_. Either 1.5, 6.25, 25 or 100 U of MNase (Worthington Biochemical) were added to pre-warmed cells and incubated at 37 °C for 3 min. Digestion was halted by moving to ice and adding 10 μl of 250 mM EDTA, 250 mM EGTA. Before DNA clean-up, the digestions were adjusted to 0.5% SDS and 10 mM Tris pH 8. For DNA cleanup, digestions were incubated with RNase (Roche) for 30 min at 37 °C, with proteinase K (Roche) for 60 min at 55 °C, and incubated at 65 °C for 60 min to reverse crosslinks. This was followed by phenol–chloroform extraction and ethanol precipitation. In some experiments, the purified DNA was subjected to a low-stringency size selection to remove high-molecular weight DNA, although excluding this step does not affect the results (data not shown). For size selection, digestion products in a volume of 100 μl of water were incubated for 5 min at room temperature with 60 μl of Agencourt AMPure XP beads (Beckman Coulter). After bead separation, the supernatant was moved to a new tube and 120 μl of new bead suspension was added. DNA was eluted from these beads and used as input into the library preparation protocol described in Bowman *et al*.^38^ Libraries were sequenced on a HiSeq2000 according to manufacturer's instructions.

For histone ChIP, after halting the MNase digestion, adding EDTA/EGTA, and adding SDS, 135 μl was removed from the 400 μl digestion as an input fraction and kept overnight at 4 °C. The remainder of each digestion was split in half (135 μl each). Each chromatin aliquot was independently adjusted to ChIP buffer conditions in a volume of 500 μl (10 mM Tris pH 8, 100 mM NaCl, 1 mM EDTA, 0.1% sodium deoxycholate, 0.5% sarkosyl, 1% Triton X-100, and 1 × COMPLETE protease inhibitors (Roche)). After adding 1 ml additional ChIP buffer to each chromatin aliquot (total volume of 1.5 ml), this was tumbled end over end for 10 min at 4 °C and then subjected to a high-speed spin in a microcentrifuge for 10 min at 4 °C. Supernatant was taken to a new tube for precipitation. Antibody (2 μl) was added to each tube (histone H3-ChIP, ab1791, Abcam; histone H4 ChIP, ab10158, Abcam). Chromatin precipitation was performed as described^38^.

### RNA-seq data generation

Drosophila S2 cells (10^7^) were harvested on ice, washed in cold PBS and their RNA was extracted using the RNeasy Kit (Qiagen) with on column DNAse digestion according to the manufacturer's protocol. Ribosomal RNA was depleted from the total RNA (5 μg) by using the RiboZero gold magnetic kit (Epicentre/Illumina) according to manufacturer's instructions. cDNA was generated with the TruSeq non–stranded kit (Illumina) according to manufacturer's instructions. cDNA Libraries for next-generation sequencing were assembled by blunt end repair and A-tailing of the cDNA, followed by adapter ligation and PCR with bar-coded sequencing primers according to Bowman *et al*.^38^ The libraries were sequenced on a HiSeq2000 according to manufacturer's instructions.

### MNase titration in human K562 cells

Human K562 cells were grown in Iscove's modified Dulbecco's medium supplemented with 10% heat-inactivated FBS at 37 °C. Human cells were expanded to yield approximately 4 million cells per reaction and crosslinked with 1.1% formaldehyde for 10 min at room temperature. Cells were lysed and nuclei were isolated using a sucrose cushion and treated with a range of 18 MNase concentrations in buffer containing 10 mM Tris pH 7.4, 15 mM NaCl, 60 mM KCl and protease inhibitors for 15 min at room temperature. EDTA and EGTA were added to stop the digestion. Cross-link reversal was performed at 65 °C for 16 h followed by RNase treatment for 30 min at 37 °C, followed by the addition of SDS to a final concentration of 1% and proteinase K digestion overnight, at 55 °C. DNA was purified by phenol–chloroform extraction and ethanol precipitation. Ampure SPRI beads (Beckman Coulter) were used in a double size selection with ratios of 0.7 × and 1.7 × to obtain a range of fragment sizes from ∼100 to 1,000 bp. The resulting fragments from four MNase concentrations in the range (5.4, 20.6, 79.2 and 304 U) were prepared individually for bar-coded sequencing^38^, on an Illumina HiSeq instrument.

### MNase titration in mouse ESCs and NPCs

J1 ESCs (ESC1) and E14-derived ESCs (ESC2) were maintained on mitomycin-C-inactivated embryonic fibroblast feeder layers in DMEM supplemented with 15% FBS (Hyclone) and 1,000 U ml^−1^ of leukemia inhibitory factor (EMD Millipore). ESC cultures were depleted of feeder cells before use in experiments. Neural progenitors (NPC1) were derived by *in vitro* differentiation from J1 ESC and maintained using previously described methods^39^. Embryonic NPCs (NPC2) were isolated from the brains of E13.5 embryos and maintained on poly-L-ornithine coated plates in DMEM:F12 containing B-27 and N-2 supplements (Gibco) and EGF+FGF-2 (10 ng ml^−1^). One million cells were crosslinked at room temperature for 10 min with 1% formaldehyde and crosslinking stopped by the addition of 125 mM glycine. Nuclei were isolated and MNase digestion performed on either 200,000 or 250,000 nuclei per reaction. Digestions were performed using 64, 16, 4 or 1 U MNase (Worthington) in 10 mM Tris pH 7.4, 15 mM NaCl, 60 mM KCl, 1 mM CaCl_2_ for 10 min (250,000 nuclei reactions) or 15 min (200,000 nuclei reactions) at 25C. Reactions were stopped with EDTA/EGTA and 0.5% SDS and 125 mM NaCl added to the samples. RNase A and proteinase K treatment along with cross-link reversal at 65 °C were performed. DNA was purified by phenol–chloroform extraction and column purification. MNase digestion was evaluated and the recovered DNA from 1 to 64 U MNase digested samples was used to generate sequencing libraries as described, without fragment length selection.

### Sequencing data alignment and processing

The sequenced paired-end reads were mapped to dm3, hg19 and mm9 genomes in the cases of *D. melanogaster*, *Homo sapiens* and *Mus musculus* data respectively using Bowtie aligner v. 0.12.9 (ref. 40). Only uniquely mapped reads with no more than two mismatches were retained. The reads with the insert sizes <50 bp or >500 bp were filtered out. Genomic positions with the numbers of mapped tags above the significance threshold of *Z*-score=7 were identified as anomalous, and the tags mapped to such positions were discarded. Read frequencies were computed in 300-bp non-overlapping bins in the case of fly data and in 500-bp bins in the case of human data for each titration point independently. The read frequencies were normalized by the corresponding library sizes to represent values per one million of mapped reads. To facilitate the comparison of the results between different genomes, the frequencies were additionally scaled by the factors representing ratios between the corresponding genome size and 100 Mb, similarly to the approach described in Kasinathan *et al*.^41^ The profiles generated for individual replicates correlated well for all titration points (Supplementary Fig. 6) and were combined into replicate sets. All major results were validated using the data generated for each replicate separately (data not shown). RNA-Seq tags were aligned using Tophat software package with default parameters^42^. RNA-Seq tag frequencies were normalized for GC content using bioconductor package EDASeq and then the expression estimates for each gene were obtained using bioconductor package DESeq^43^,^44^. Assessment of statistical significance, K-means clustering, HHM generation and other analyses were performed in R programming environment (http://r-project.org).

### Annotated regions and external data sets used in this study

The coordinates of the genes were taken according to the annotations for dm3, mm9 and hg19 versions of the fly, mouse and human genomes, respectively. The coordinates of the active enhancers were taken according to modENCODE annotation for S2 cells^27^ and according to a recent study by Hnisz *et al*.^34^ for mouse ES cells. Gene expression profile for K562 cells was downloaded from ENCODE data repository (http://genome.ucsc.edu/ENCODE). The fly genome annotation based on the combinatorial patterns of chromatin modifications (‘chromatin states') was taken as defined by modENCODE consortium^45^. Coordinates of the topological domains in fly S2 cells were taken from Ulianov *et al*.^31^. To ensure robust domain definition and classification, another set of topological domains was used (TADs identified in fly embryos^32^). Only those S2 domains that overlapped with the embryo domains of one epigenetic class (‘Active', ‘HP1-centromeric', ‘PcG', or ‘Null') were selected for the analysis. The data on nucleosome density, salt fractionation, histone modifications and variants, as well as regulatory protein binding were obtained from mod/ENCODE repositories (http://genome.ucsc.edu/ENCODE and http://intermine.modencode.org). The coordinates of Psc and Trx binding sites were identified by Enderle *et al*.^46^ The data on DNA accessibility measured with help of DNA methylation assay (MeDIP) in S2 cells were obtained from the study by Bell *et al*.^29^

### Calculation of MACC

MACC values were obtained for each 300-bp (fly) or 500-bp (mouse and human) bin in the genome by fitting linear regression on the normalized read frequencies computed for each titration point (four frequencies per bin, Fig. 1a). In the fitting procedure we used log-scale for the MNase concentrations, which allowed us to preserve similar distances between the consecutive MNase points. The bins that had no mapped reads at any of the titration points were excluded from the consideration. To address possible bias in MACC values associated with different GC content of the underlying DNA sequence we applied a correction procedure based on a locally weighted scatterplot smoothing (LOWESS) as implemented in Bioconductor package limma (Supplementary Fig. 18). In the case of human data the LOWESS correction was performed separately for the bins that were located either within or outside CpG islands to account for the specific sequence composition of these genomic elements. To confirm that GC-content normalization does not lead to appearance of artifacts in our results, we verified that the observed trends in MACC distribution can be detected for non-GC-corrected values (Supplementary Fig. 18). We note that for the uncorrected values the trends are somewhat less pronounced, which underscores the importance of GC correction applied to MACC values in this study. MACC values for H3/H4 ChIP and ChIP input data were computed in the same way as above.

The MACC scores were validated with additional computational analyses and experiments described below. To test how significance of the linear fit of the tag frequencies in each bin affects the results we used adjustment of the MACC scores using *P* values computed for either Pearson correlation or Mann–Kendall trend test. The MACC values were adjusted by the factor equal to (1−*P* value) so that the bins with high *P* values (low significance) would have near-zero weighted MACC scores. The MACC scores computed with and without correction correlated well (*r*=0.96 and higher); thus this correction had only a limited effect on the results and was not used for final scores. To test how the number of used titration points affects the MACC scores we repeated MACC estimation for individual replicates using 4, 3 and 2 titration points. This analysis confirmed that using complete set of the titration data (4 points) results in the strongest correlation between replicates (Supplementary Table 2). To validate our approach further, we performed additional measurement of MACC values in S2 cells cultured separately from the S2 cells used for most analyses in this study. Comparison of the two MACC profiles (‘additional' MNase-Seq data set versus ‘main' MNase-Seq data set) showed similar numbers of sequenced tags (Supplementary Table 1) and substantial correlation (Supplementary Fig. 18). Also, both profiles showed similar patterns of enrichment in the annotated genomic regions (Fig. 2c; Supplementary Fig. 18). Finally, to ensure that the obtained results on chromatin accessibility are not a function of the next-generation sequencing platform used in this study, we preformed MNase-qPCR experiment for a number of genomic locations. In this way we validated the sites that have either positive or negative MACC values and are associated with either histone or non-histone–DNA protection (Supplementary Figs 12, 19 and 20).

### Analysis of MACC profiles

To facilitate comparison with MACC profiles, the signals for all external markers were re-computed for the same 300-bp bins as those used for MACC computations. The profiles around specified sets of sites were computed by using linear interpolation of MACC values associated with 300-bp bins and the resulting average profiles were additionally smoothed in the 40-bp running window. Two-state HMM and Viterbi algorithm were used to map chromatin accessibility states based on MACC profiles. The computations were performed using R-package RHmm (https://r-forge.r-project.org/projects/rhmm/). For generation of HMM based on randomized data, shuffling of MACC profile within each chromosome was used. Hierarchical clustering was performed using unweighted pair group method with the distance between profiles computed as (1—Pearson's correlation coefficients). To ensure that our findings are not biased by variability in the fragment size distributions, we reproduced crucial observations with MNase and MACC profiles computed only with fragments of mono-, di- and tri-nucleosomal lengths (Supplementary Figs 21–25).

### Selection of TSS proximal regions

The TSS proximal regions were defined as ±1 kb around TSS in the case of fly data and ±2 kb in the case of mouse and human data. TSS proximal regions overlapping with other genes were excluded from consideration. Additionally the bins that overlapped with enhancer regions were filtered out (such bins were retained in the analysis of MACC values at enhancers).

### Identification of protein binding sites

NHP-binding data produced by modENCODE consortium and individual studies (ChIP-chip and ChIP-Seq) were collected from public sources (as described above). The protein enrichment, that is, ChIP signal over input, was computed in 300-bp bins for each of collected profiles. The enrichment was further transformed into *Z*-scores. Bins with *Z*-score above a given threshold were selected as NHP-binding sites. When a particular protein was represented by more than one profile we preserved the profile with lowest variability in the number of peaks obtained under different *Z*-score thresholds (for *Z*-scores in (2,..,5)).

### Comparison of c-MACC and h-MACC profiles

To facilitate comparison, the c-MACC and h-MACC profiles were median shifted so that the median values in their genome-wide distribution were equal to zero. The following thresholds were used to place bins in group 1 (high c-MACC and low h-MACC) and group 2 (high c-MACC and high h-MACC): c-MACC higher than 80% of all positive c-MACC scores and h-MACC lower than 10% of all positive h-MACC scores (group 1); c-MACC higher than 80% of all positive c-MACC scores and h-MACC higher than 90% of all positive h-MACC scores (group 2). MACC peaks represent bins with c-MACC values higher than those in the adjacent bins (c-MACC_i−1_<c-MACC_i_>c-MACC_i+1_). H3 enrichment was computed in 300-bp bins as an H3-ChIP/input ratio between fragment frequencies pooled over all MNase titration points. To further validate MACC peak features we also performed c- and h-MACC comparison at higher resolution using 100-bp bins, and this analysis reproduced our major findings (Supplementary Figs 10c, 26). To estimate expected overlap between group 1 (group 2) and NHP-binding sites, the positions of the MACC peaks were randomized separately for each group preserving their total number. The expected value of overlap represents average over 10 independent randomizations. The enrichments of MACC peak occurrence in the annotated regions of the genome were computed as ratios between the fractions of the bins that overlap a given class of regions (for example, promoter, TES, and so on) and the fraction of the genome covered by this class of regions. Region definition was used as described above.

## Additional information

**Accession codes:** Sequencing data used in this paper were deposited into NCBI Gene Expression Omnibus (GEO) under accession number GSE78984.

**How to cite this article:** Mieczkowski, J. *et al*. MNase titration reveals differences between nucleosome occupancy and chromatin accessibility. *Nat. Commun.* 7:11485 doi: 10.1038/ncomms11485 (2016).

## Supplementary Material

Supplementary Information Supplementary Figures 1-26 and Supplementary Tables 1-2
