# Supplementary material for: MNase titration reveals differences between nucleosome occupancy and chromatin accessibility
Source: Nat Commun. 2016 May 6;7:11485. doi: 10.1038/ncomms11485 (PMC4859066; doi:10.1038/ncomms11485)
Supplement: Supplementary Information — Supplementary Figures 1-26 and Supplementary Tables 1-2 [file ncomms11485-s1.pdf]

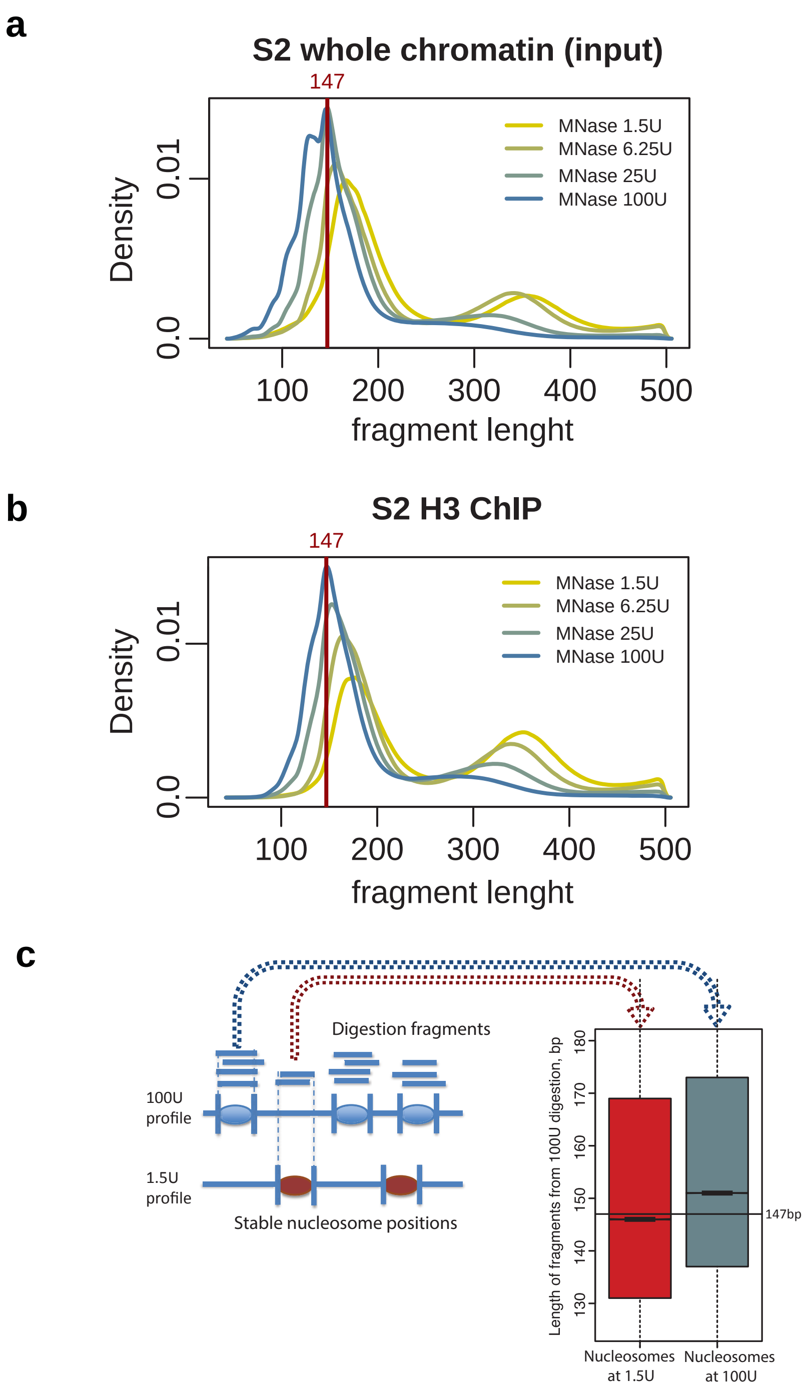

Supplementary Figure 1.
Characteristics of digestion fragments in S2 cells.
(a,b) Distribution of the digestion fragment lengths in input (a) and H3 ChIP (b) libraries generated for S2 cells. **(c)** Nucleosomes detectable at light digestion condition protect loci of subnucleosomal sizes in deep digestion. Fragments produced in 100U digestion which are related to the stable nucleosome positions identified for either 1.5U profile (red) or 100U profile (blue) were compared in terms of their lengths. The fragments associated with 1.5U nucleosomes exhibit shorter lengths, with median being smaller than the expected nucleosomal size of 147bp.

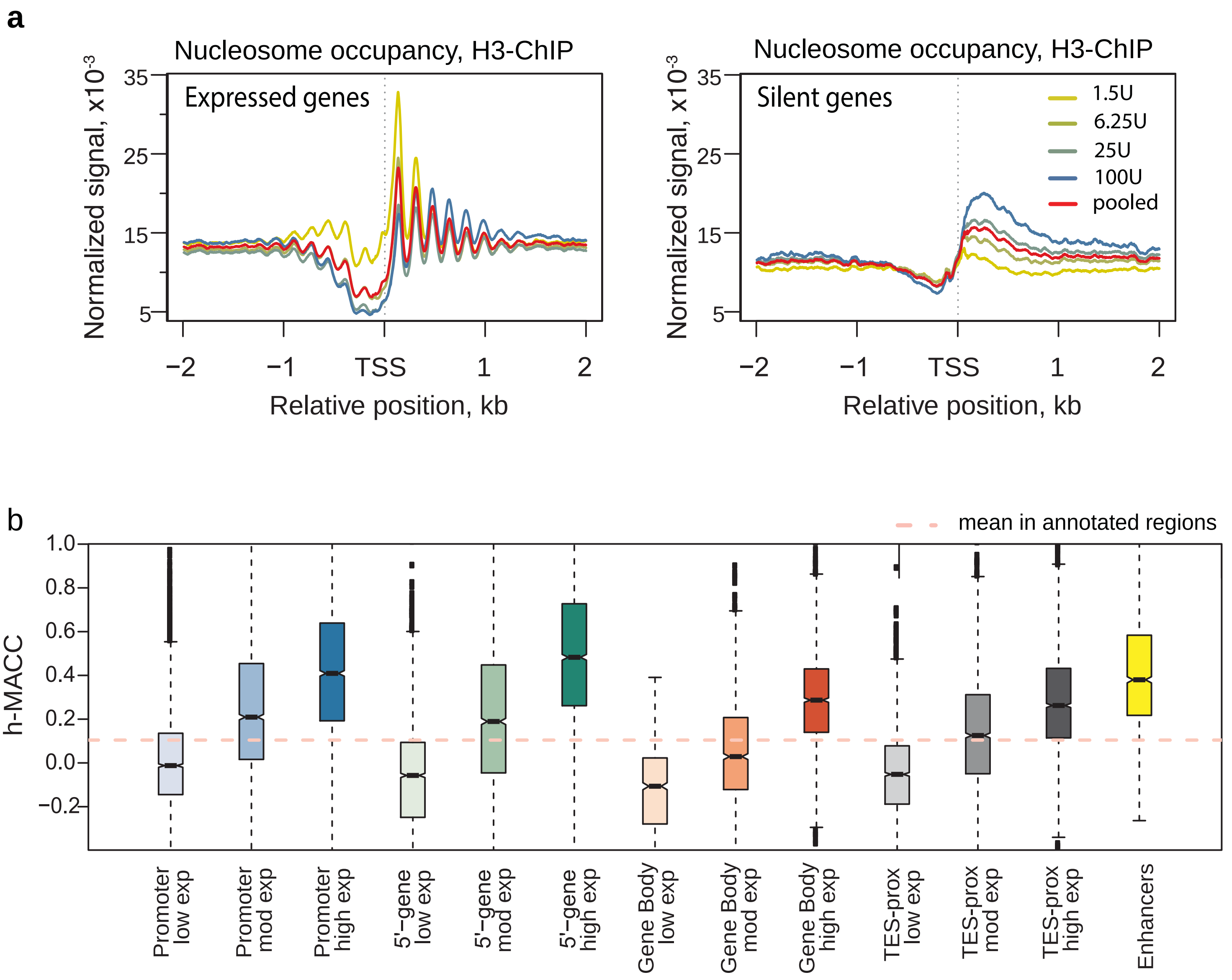

Supplementary Figure 2.
Analysis of H3 ChIP data.
(a) MNase-seq profiles around TSS (transcription start sites) for expressed (left) and silent (right) genes. Yellow-blue color scheme indicates MNase concentration levels (1.5, 6.25, 25 and 100 U/uL), with bright yellow corresponding to the lowest concentration and dark blue corresponding to the highest. The red lines depict the pooled profiles. **(b)** Distribution of h-MACC values within annotated regions. The results are shown for promoters (1kb upstream of TSS, blue), 5'-ends of genes (1kb downstream of TSS, green), gene bodies (red), regions around transcription end sites (around +/-1kb of TES; grey), and enhancers (identified by modENCODE consortium for S2 cells, yellow). The shade of the color within each group of regions encodes the magnitude of expression level. Pink dashed line represents the mean for h-MACC value within annotated regions. For the overlapping regions the following priority rule was used: enhancer > promoters > 5'-gene > TES-prox > gene bodies.

a

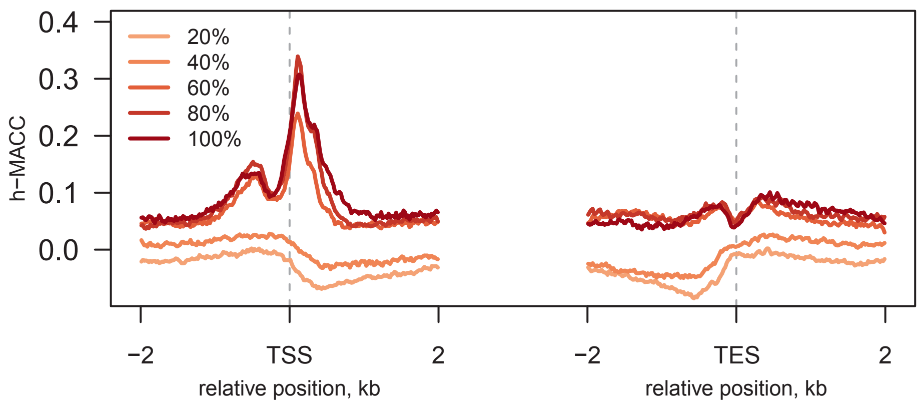

b

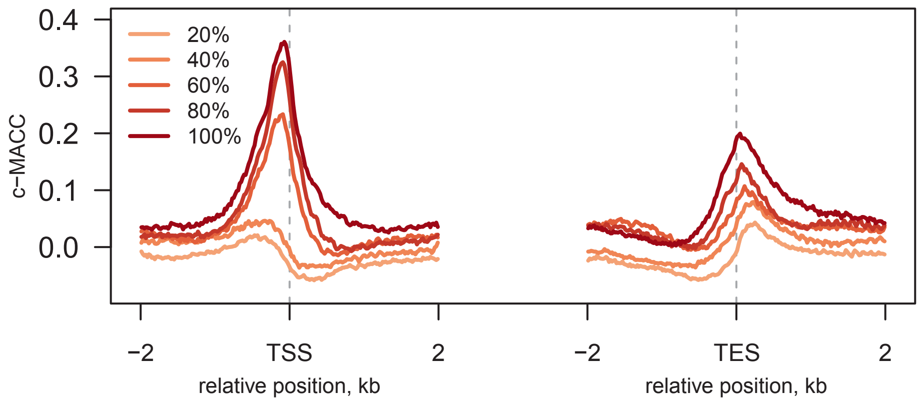

Supplementary Figure 3.
Accessibility profiles around transcription start sites (TSS) and transcription end sites (TES).
(a) h-MACC and **(b)** c-MACC. The profiles are shown for quintile groups based on gene expression levels, color-coded from the light orange (lowest expression) to dark red (highest expression).

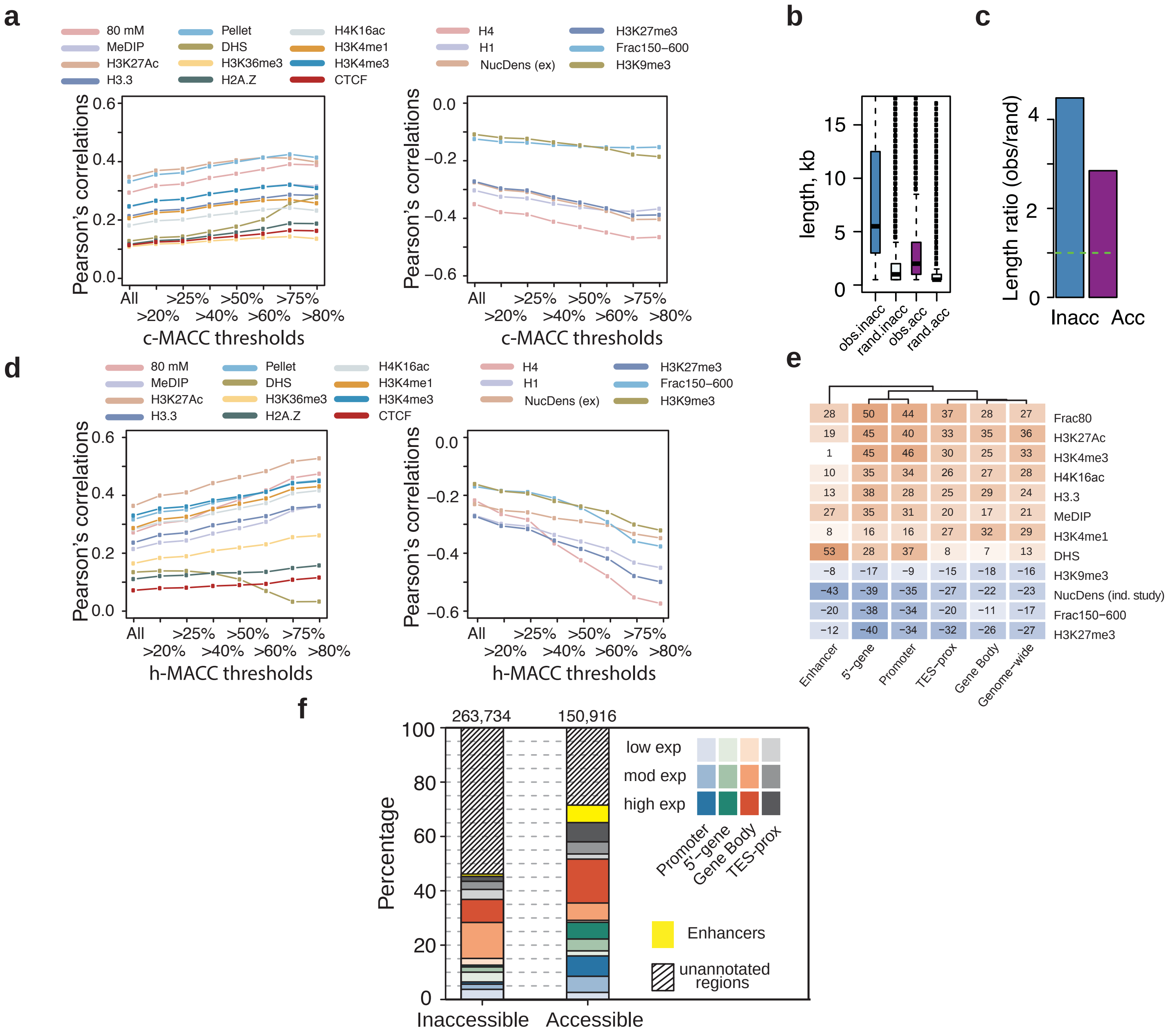

Supplementary Figure 4.

Correlation between c-MACC and h-MACC with other metrics of chromatin structure.

(a) Correlations of c-MACC with chromatin markers were computed for all bins ("All") and the bins characterized by c-MACC values above specified thresholds (higher than 20% ... 80% of all absolute values). Left panel presents positive correlations and right panel presents negative correlations. **(b)** Comparison of the length distributions of continuous stretches of inaccessible and accessible HMM c-MACC states. The distributions of c-MACC states based on actual data (dark blue and purple boxes) are compared to the HMM segmentation based on randomized distributions c-MACC values (light blue and pink boxes). Blue boxplots correspond to lengths of inaccessible states and purple boxplots to lengths of accessible states. **(c)** Ratios of the average lengths of continuous stretches of HMM states computed for observed and randomized c-MACC profiles. **(d)** Correlations of h-MACC with physical properties of chromatin computed as in (a). **(e)** A heatmap depicting relation between h-MACC and physical properties of chromatin computed within annotated regions. The values appearing in the heatmap cells represent Pearson's correlation coefficients multiplied by 100. Color scale encodes the same values, with red and blue colors standing for positive and negative correlations respectively. The heatmap was clustered by columns. **(f)** Distribution of h-MACC states in genomic regions. Accessible and inaccessible states were identified with HMM for 300-bp bins. Stacked bars represent fractions of the bins assigned to each state in the corresponding regions. The numbers of bins in each state are shown above the bars.

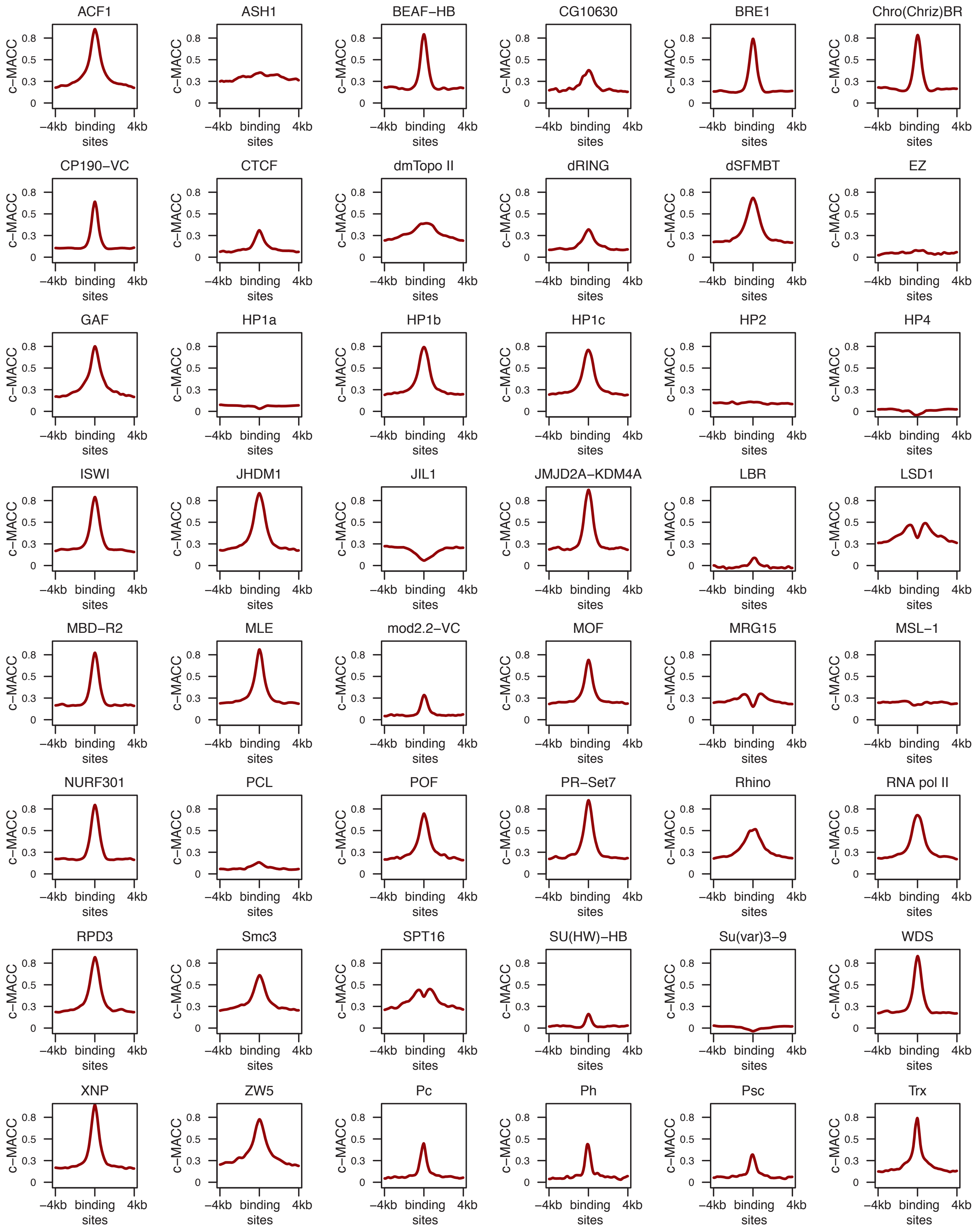

Supplementary Figure 5.
c-MACC profiles around binding sites of selected proteins.
Protein binding data were either generated by modENCODE consortium or taken from Enderle et al. (Genome Res. 2011 Feb; 21(2):216-26). To identify binding sites, the enrichment z-scores were computed in 300bp bins genome-wide. Bins with z-score above 3 were selected as binding sites.

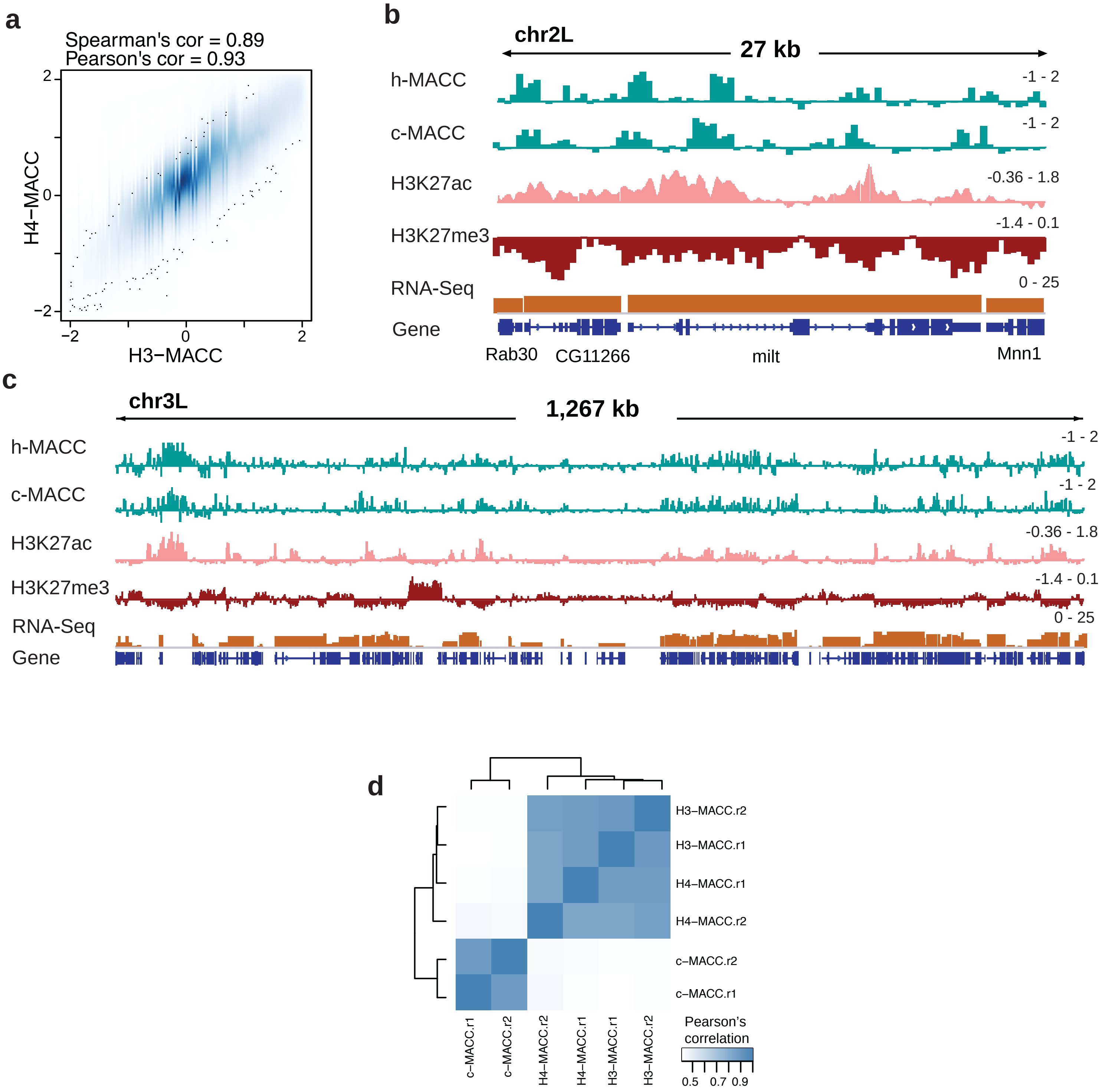

Supplementary Figure 6.
Similarity between c- and h-MACC.
(a) Correlations of MACC scores based on histone H3 and H4 chromatin immunoprecipitation assays (H3- and H4-MACC respectively) and MACC computed for whole chromatin (c-MACC). All MACC profiles were computed for two independent replicates. **(b,c)** Examples showing similarity of h-MACC and c-MACC accompanied by H3K27ac, H3K27me3 and gene expression at a ~27-kb (b) and a ~1,267-kb (c) loci. **(d)** Correlation of the H3- and H4-MACC profiles computed for individual replicates.

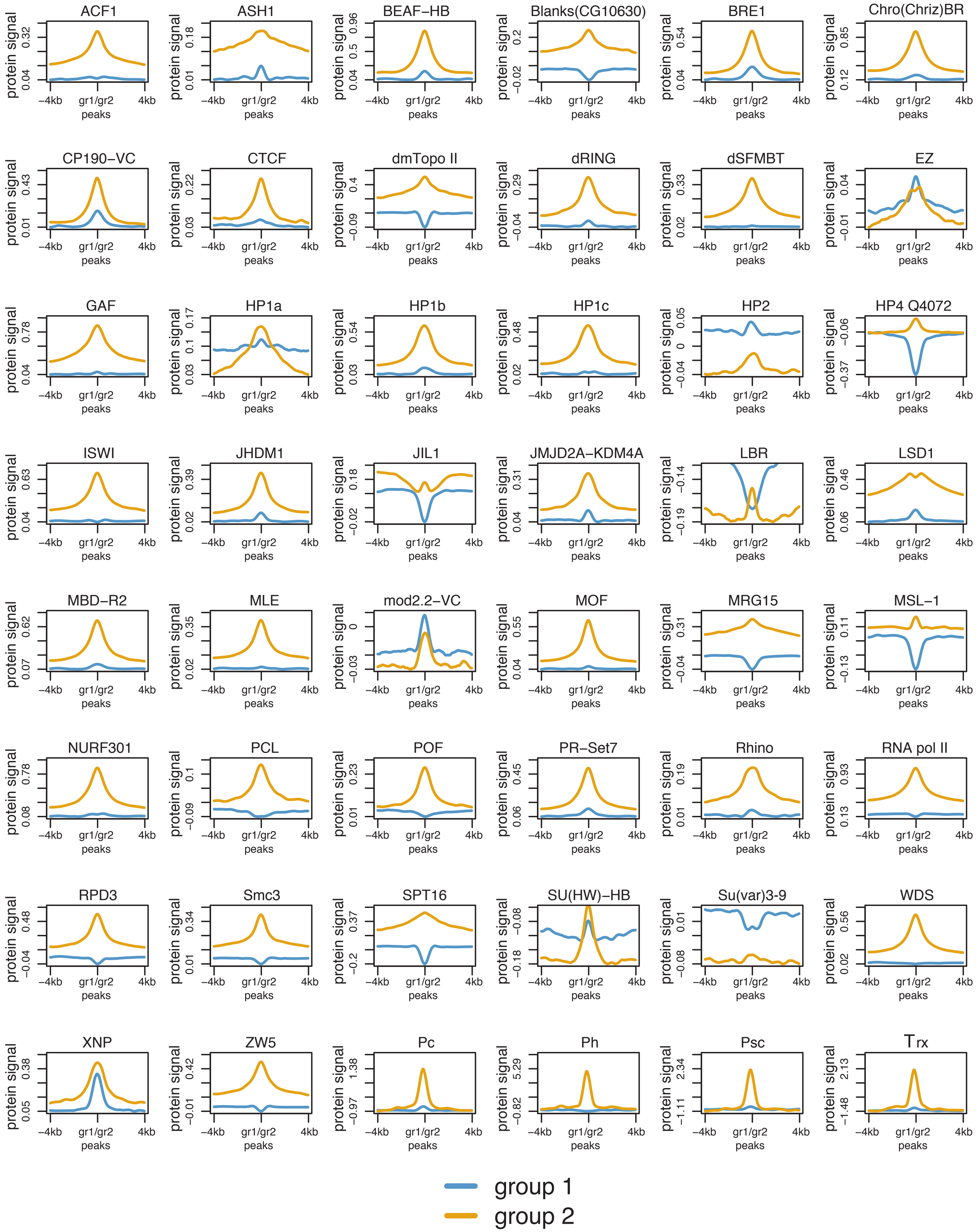

Supplementary Figure 7.

Profiles of protein binding signals around bins characterized as group 1 (blue) and group 2 (orange). Protein binding data were either generated by modENCODE consortium or taken from Enderle et al. (Genome Res. 2011 Feb; 21(2):216-26).

a

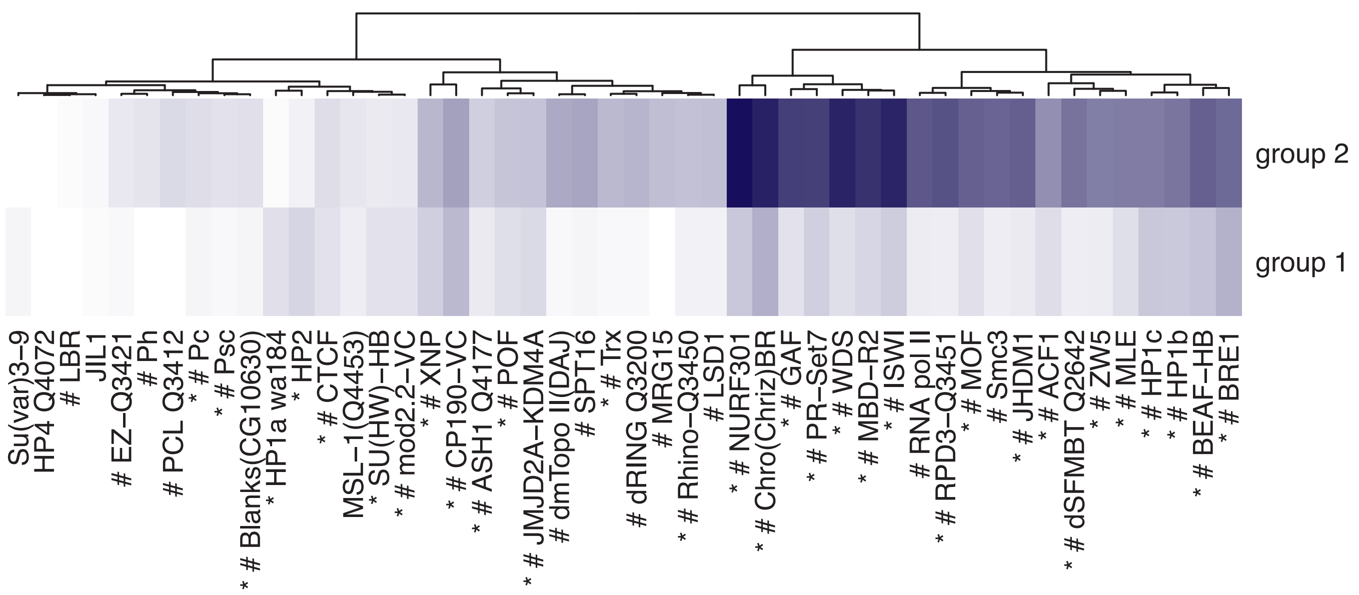

b

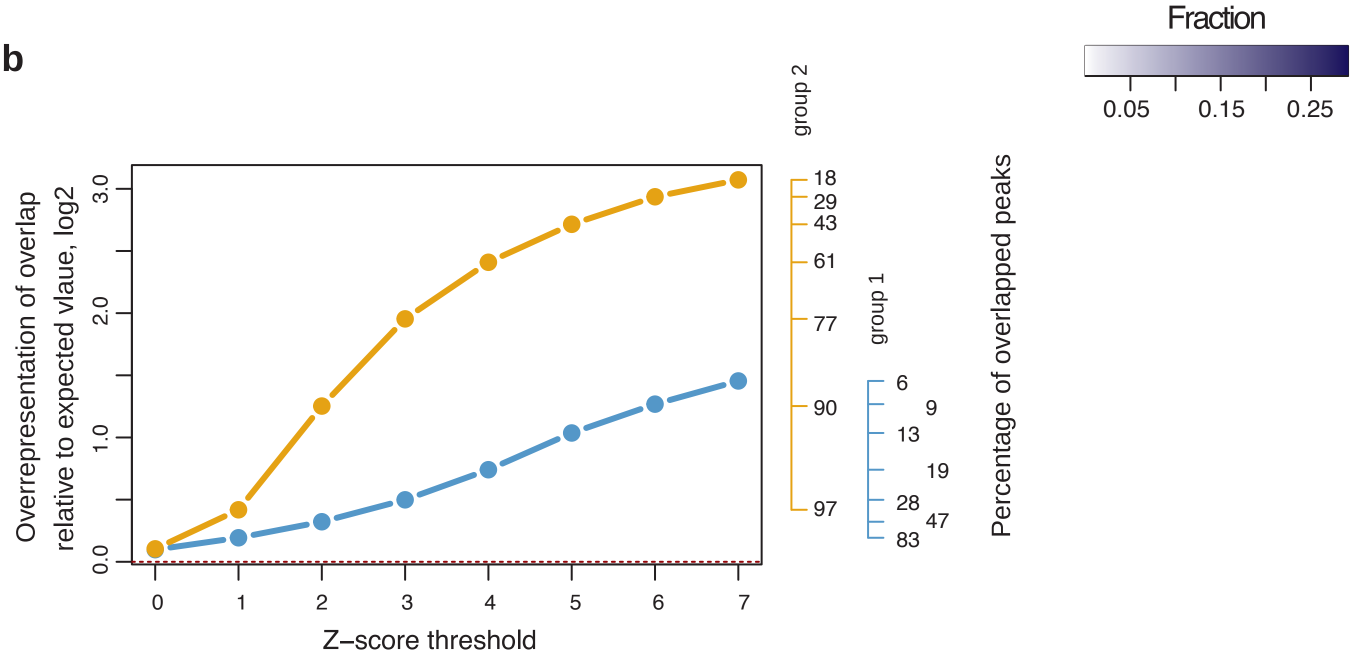

Supplementary Figure 8.
Overlap of protein binding sites with the loci from group 1 or group 2.
(a) Heatmap shows fraction of the loci from group 1 and group 2 overlapped by the binding sites of each protein included in the analysis (modENCODE data on protein enrichment was used. Z-score = 3 was used as a threshold to identify protein binding; see Methods for more details). The asterisk and hash tag symbol indicate significance of the overlap of the protein binding sites with MACC peaks from group 1 and 2 respectively ($P<0.05$, Fisher's exact test) **(b)** Overlap of protein binding sites with the MACC peaks from group 1 or group 2 (blue and orange lines respectively). The overrepresentation values appearing on the Y-axis on the left side of the plot correspond to the ratio of the percentage of the MACC peaks overlapped by a binding site of at least one protein included in the analysis (numbers on the right side of the plot) to the percentage expected for randomized distribution of the sites (see Methods for the description of the data used in the analysis). The X-axis provides the threshold values used to call protein-binding sites. Red horizontal line gives the level of 'no overrepresentation over random'. Notice the monotonic increase in overrepresentation level upon increase in binding site threshold, which indicates that more significant protein-binding sites have higher probability to overlap MACC peaks.

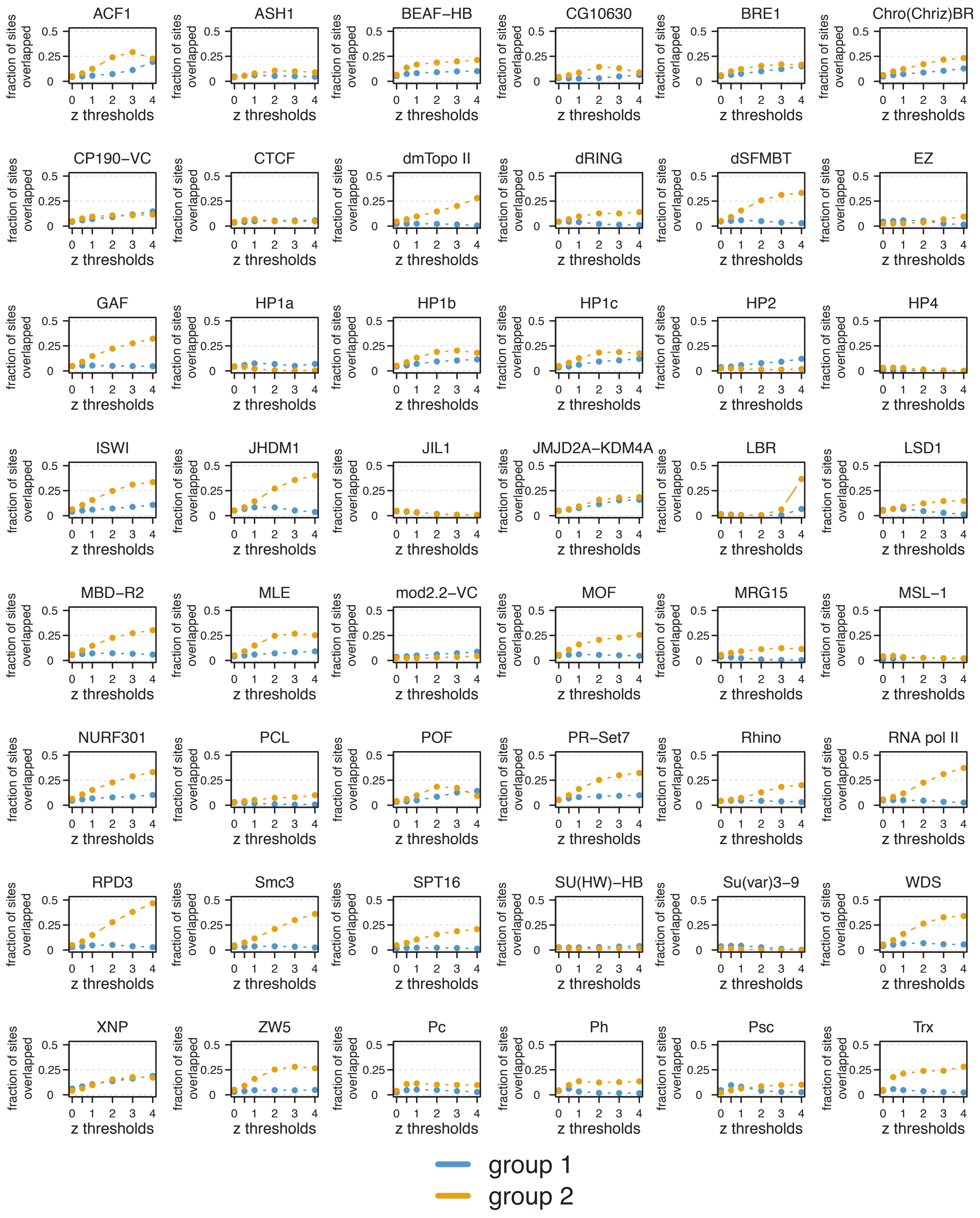

Supplementary Figure 9.

Fractions of the bins from group 1 and group 2 overlapped by protein-binding sites of selected proteins.

Each plot corresponds to an individual protein. X-axis stands for Z-score thresh- old used to obtain a set of protein binding sites and Y-axis stands for fraction of bins from group 1 or group 2 overlapped by the obtained set. Blue and orange lines correspond to group 1 and 2 respectively.

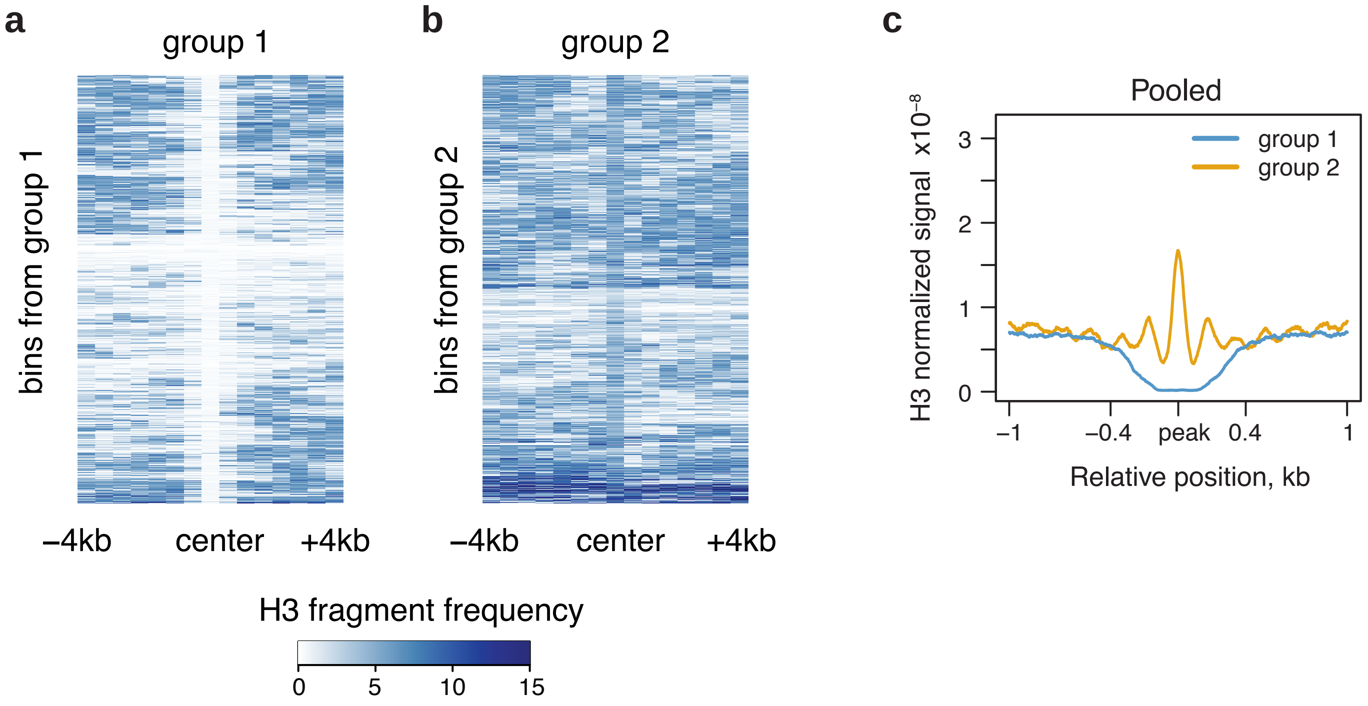

Supplementary Figure 10.
Characteristics of bins assigned to group 1 or group 2.
(a,b) Heatmaps showing H3 fragment frequencies around bins from group 1 (a) and group 2 (b). All bins from each group, peaks and not peaks were included in this analysis. Notice that H3 frequency is close to zero for most of the loci from group 1, while is it considerable for the loci from group 2. **(c)** Pooled digestion profiles of H3 occupancy around group 1 and group 2 peaks computed as in Fig. 3, but with 100 bp resolution

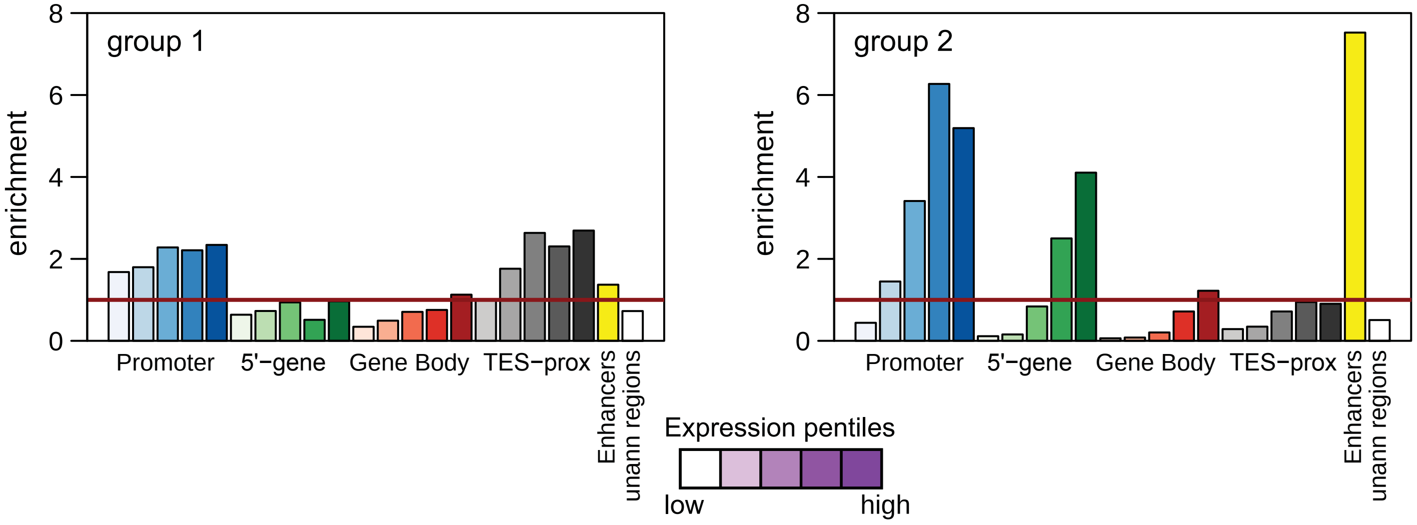

Supplementary Figure 11.
Genomic distribution of the MACC peaks from group 1 or group 2.
The enrichment values were computed relative to the expected values for each type of the genomic regions. When applicable, the genomic regions were stratified by gene expression (pentiles). See Methods for region definitions. The horizontal red lines provide reference of 1 (‘no enrichment’).

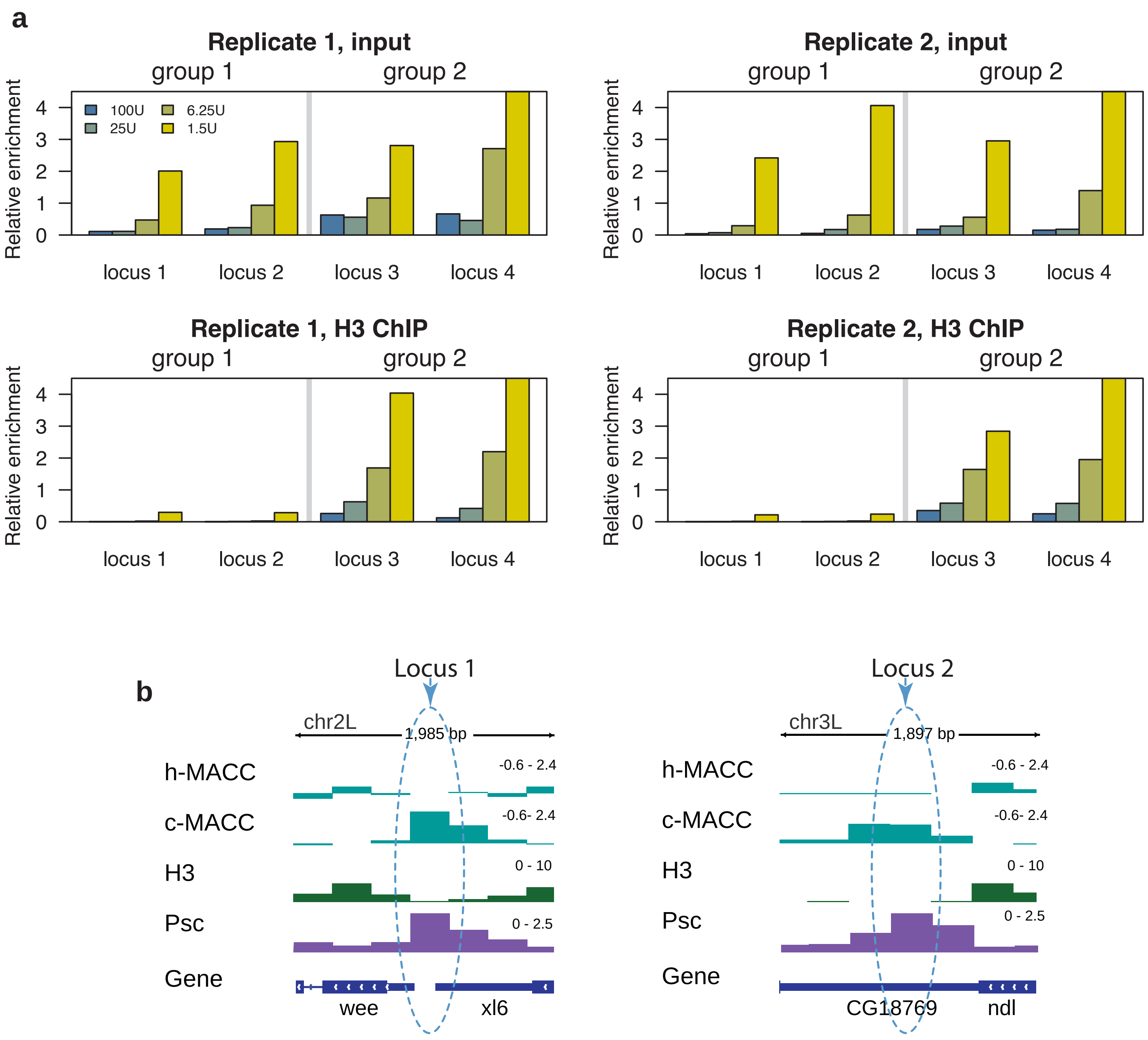

Supplementary Figure 12.
qPCR validation of regions identified as group 1 or group 2.
(a) qPCR validation of the loci identified as group 1 or group 2. Loci 1 and 2 represent sites from group 1 and loci 3 and 4 represent sites from group 2. The results are reported for two biological replicates. The enrichment values (y-axis) were computed relative to genomic DNA. Decrease in qPCR signal upon increase in the MNase concentration is equivalent to positive MACC values. Thus, the qPCR results are consistent with hand c-MACC values obtained in genome-wide profiles (MNase-Seq). **(b)** Screen-shots illustrating the distributions of MACC, H3 occupancy and protein binding at the selected group 1 loci. We note that group 1 sites overlap with Psc binding sites but do not have H3 presence.

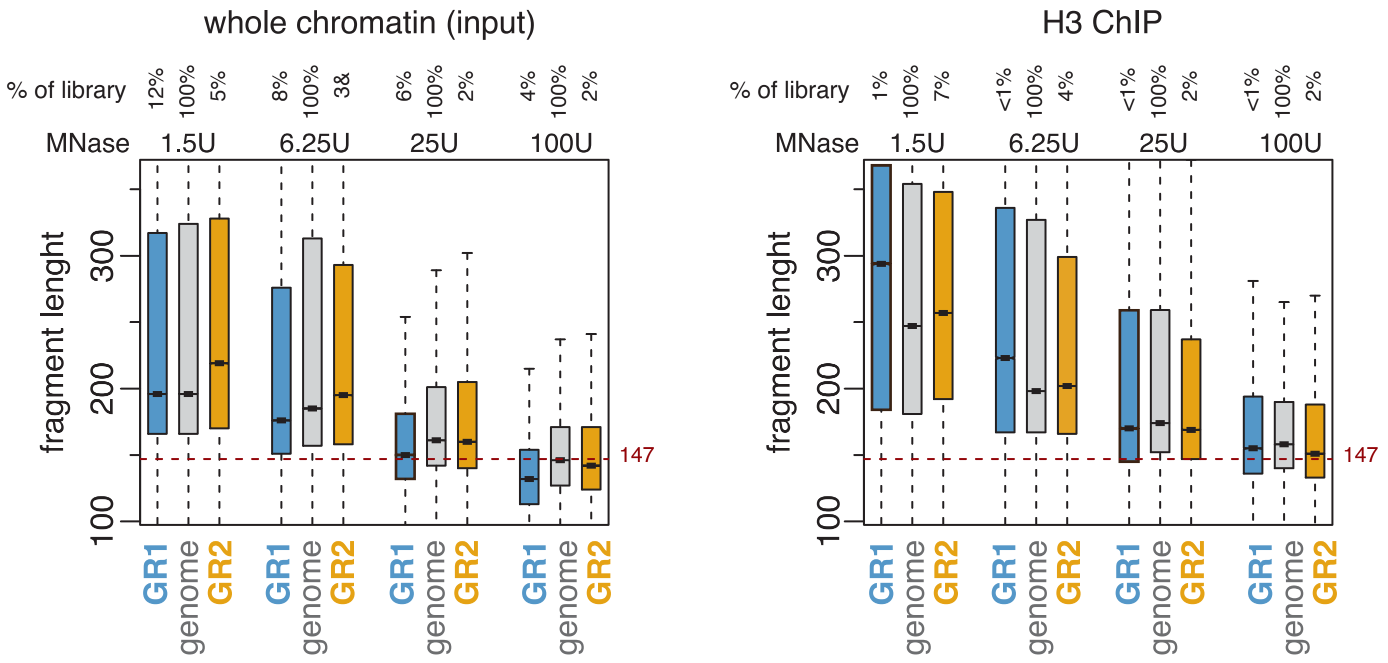

Supplementary Figure 13.
Distributions of fragment lengths in individual digests at the sites from group 1 and group 2.
The genome-wide fragment length distributions are shown for comparison. Left panel corresponds to input libraries, and right panel to H3 ChIP libraries. Percentages of the fragments at the sites from group 1 and group 2 are shown above the corresponding boxplots.

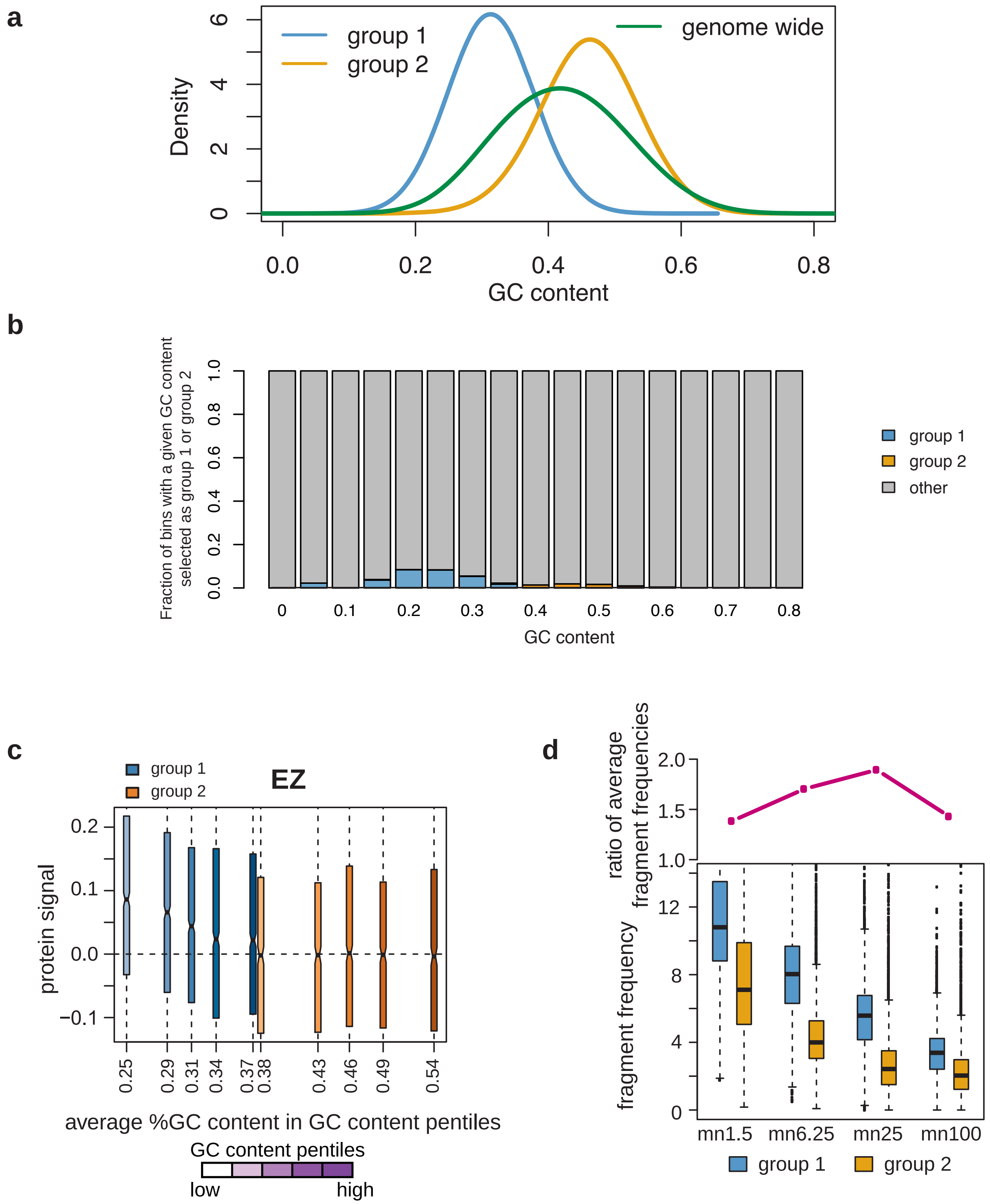

Supplementary Figure 14.
Sequence analysis for group 1 and group 2 sites.
(a) Distributions of the GC-content values for the sites from group 1 (blue), group 2 (orange), and genome-wide (green). We note that while the distributions are different they substantially overlap. **(b)** Stacked barplot representing the fractions of the sites with a specified GC-content assigned to group 1 or group 2 (blue and orange parts of the bars respectively); the grey part of each bar represents the sites not assigned to either group. **(c)** An example of the protein (Ez) that is stronger enriched at group 1 sites with low GC-content. **(d)** The sequence bias in the ratios of average fragment frequencies at sites of group 1 and group 2 is not a monotonic function of the digestion level.

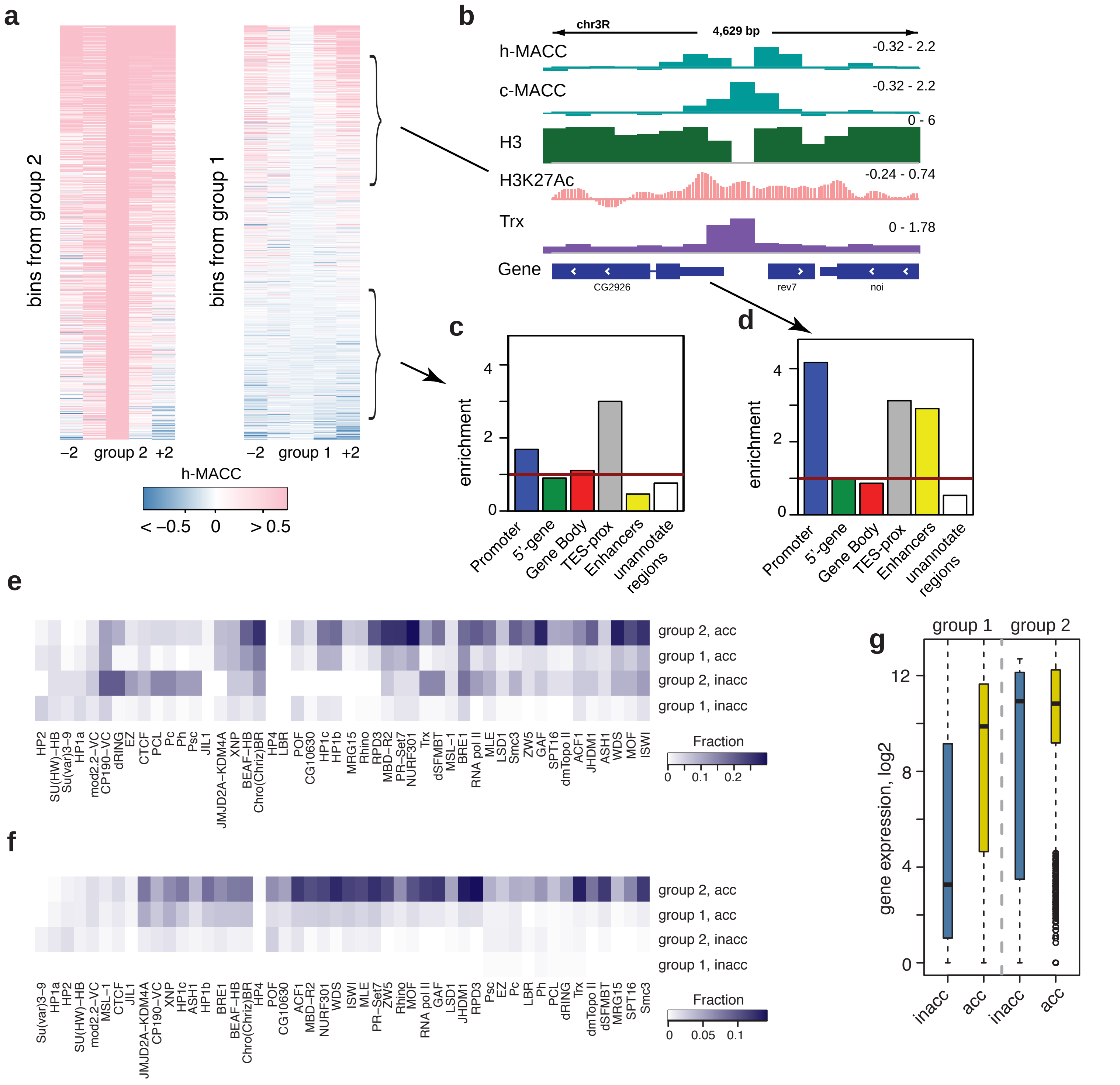

Supplementary Figure 15.
Fine structure of chromatin around peaks from group 1 and group 2.
(a) Heatmap representation of h-MACC scores around bins from group 1 (left) and group 2 (right) -2 and +2 stand for bins preceding and following selected bins respectively. Bins adjacent to group 2 sites are predominantly accessible (positive h-MACC), while bins adjacent to group 1 sites show more diverse patterns. **(b)** Example of group 1 bin that reflect binding of Trx, flanked by two accessible nucleosomes (note h-MACC peaks and H3 build-up encompassing Trx binding site). **(c,d)** Genomic distribution of group 1 peaks flanked by inaccessible (c) or accessible (d) positions. **(e)** Heatmap visualization of the fraction of peaks from group 1 and group 2 overlapped by binding sites of selected proteins; "inacc" and "acc" stand for bins encompassed by inaccessible and accessible bins respectively. Selection of protein binding data and identification of their binding sites were performed as described in Methods. **(f)** Heatmap visualization of the fraction of protein binding sites overlapped by peaks from group 1 and group 2. **(g)** Expression of genes whose TSS proximal regions [TSS-1kb,TSS+1kb] are overlapped by the regions corresponding to the subsets of group 1 or group 2 (encompassed by inaccessible or accessible bins).

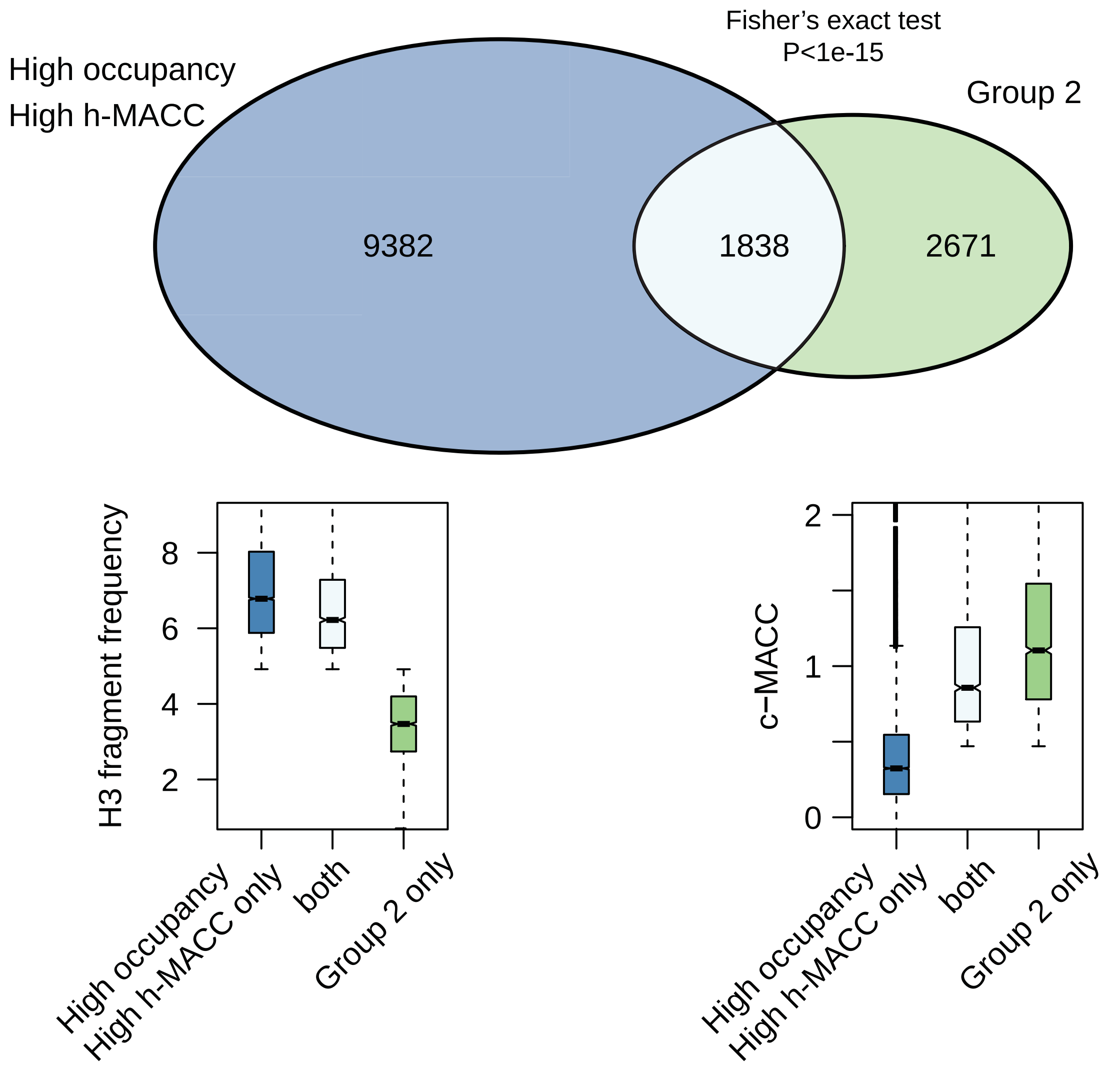

Supplementary Figure 16.
Comparison of MACC peaks from group 2 with the regions of high occupancy and high accessibility (cf. Figure 4a).
The first set (green oval) was identified with h- and c-MACC values, while the second set (blue oval) was identified with H3 fragment frequencies and h-MACC values. Venn diagram shows significant overlap (P<1e-15) between selected sets, and the boxplots show distributions of H3 fragment frequencies (left) and c-MACC (right) in the compared sets.

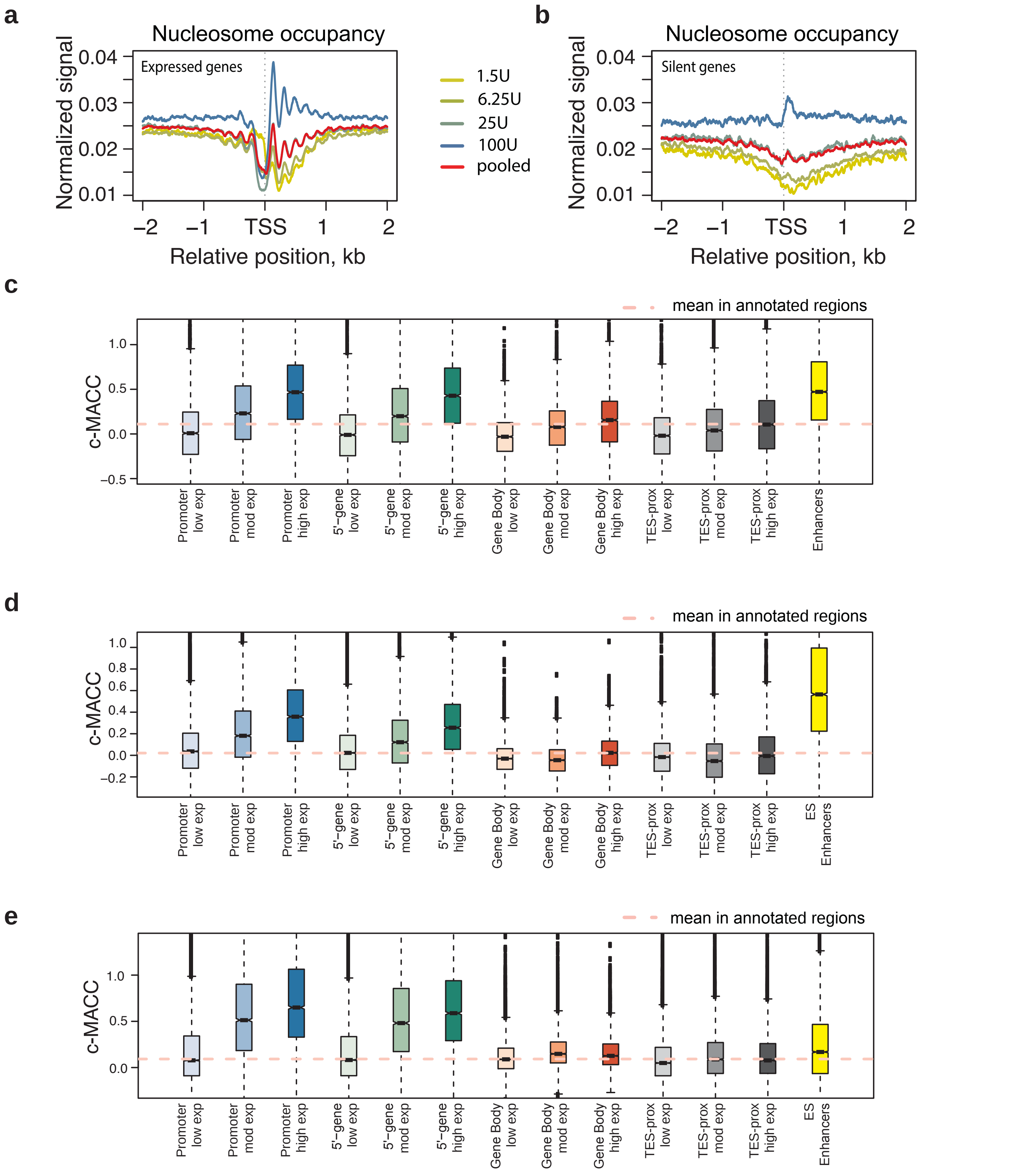

Supplementary Figure 17.

c-MACC profiling in the human and mouse genomes.

(a,b) MNase-seq profiles around TSS for expressed (a) and silent (b) genes in human genome. Blue-yellow color scheme indicates MNase concentration levels, with dark blue color corresponding to the highest and bright yellow to the lowest concentration. **(c-e)** Distribution of c-MACC values within annotated regions in K562 cells (c), mouse ES cells (d) and mouse NP cells (e).

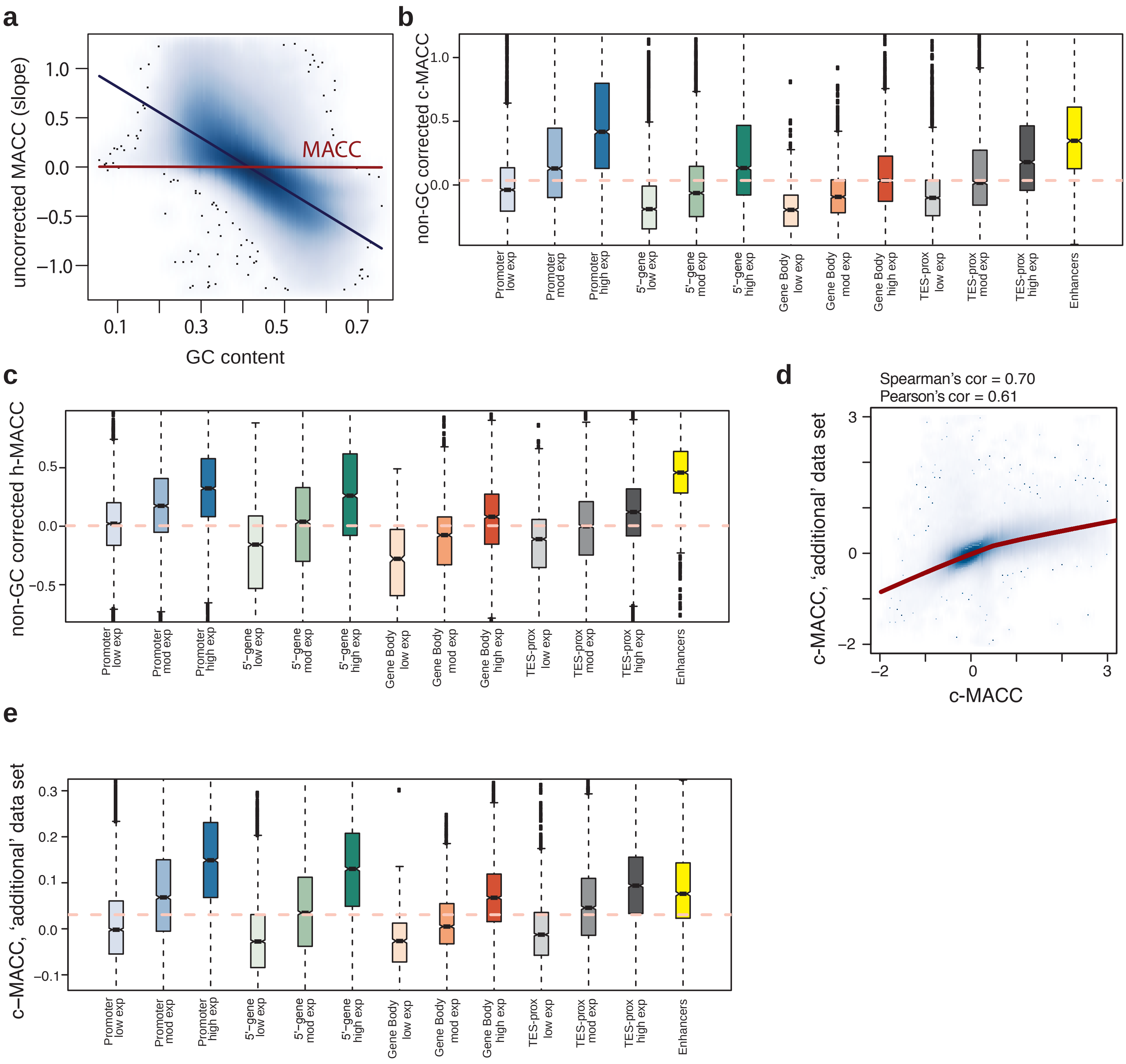

Supplementary Figure 18.
Comparison of data sets.
(a) Correction of GC-bias in the linear regression slopes computed for non-overlapping 300-bp bins. The LOW-ESS-based correction was performed on genome-wide distribution of slope values (blue) to remove the dependence of these values on the GC-content of underlying DNA sequence. The blue and red lines represent correlation trends in uncorrected and corrected data, respectively. **(b,c)** Distribution on non-GC corrected MACC values within annotated regions (for details see main text Figure 2 legend). **(d)** Comparison of the correlation of MACC values obtained in two MNase-Seq experiments performed for the sub-lines of S2 cells (main data set used in this study and 'additional' data set). (e) Distribution of c-MACC values computed for 'additional' data set within annotated regions.

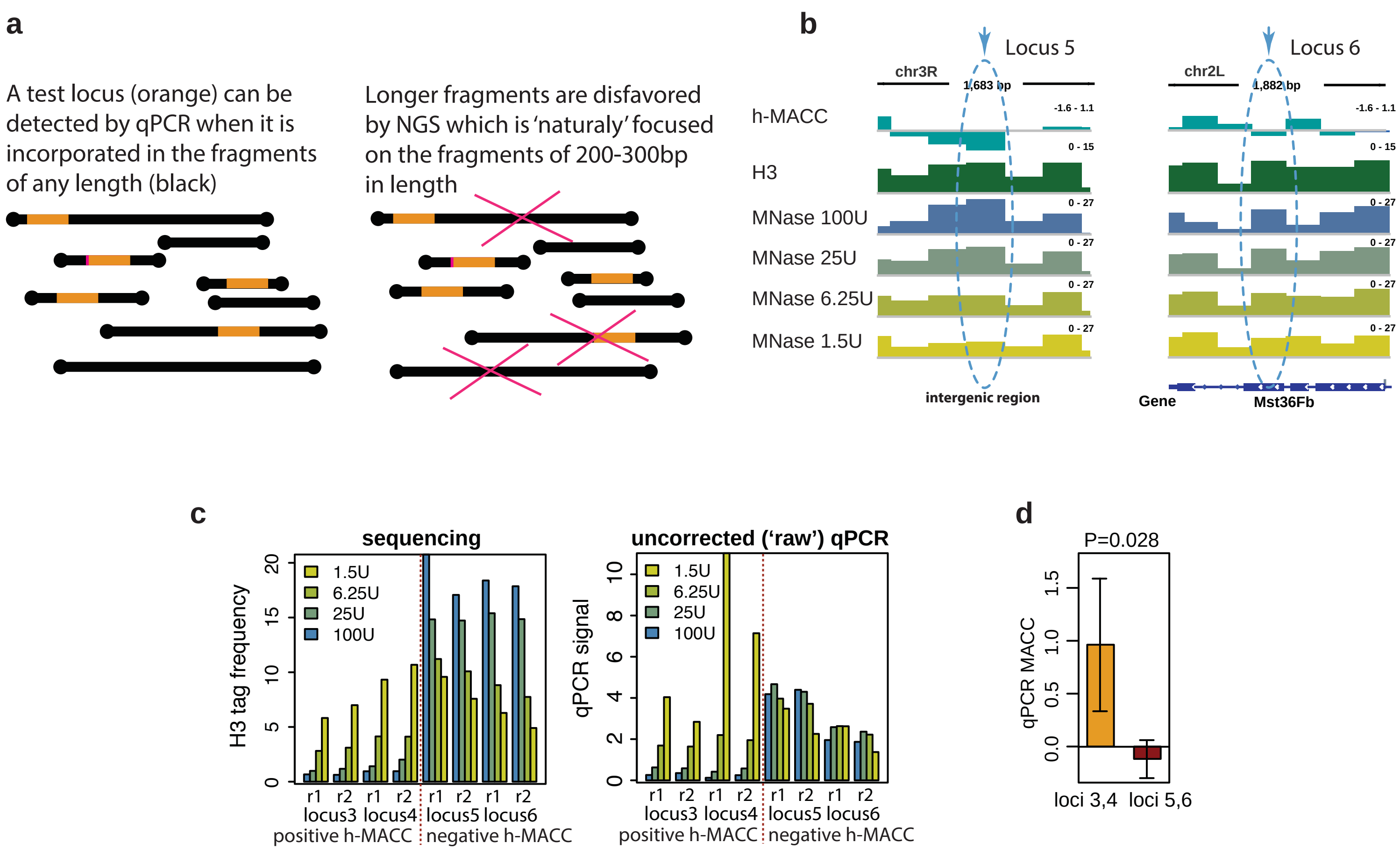

Supplementary Figure 19.

qPCR validation of the loci of high and low nucleosome accessibility (positive and negative h-MACC respectively).

(a) Schematic illustration of the differences between qPCR and next generation sequencing (NGS) approaches as applied to MNase-based assays. qPCR detects test sequence embedded in both long and short digestion fragments, while NGS tends to select against long fragments. Thus, the detected signal of qPCR and NGS is expected to differ. Specifically, the sites of low accessibility (negative MACC) would yield similar numbers of fragments (of gradually decreasing lengths) at all MNase concentrations, which would contain the tested negative MACC site. This would result in roughly 'flat' profiles at negative MACC sites across MNase concentrations for qPCR. **(b)** Browser snapshots illustrating the 'negative h-MACC sites' (loci 5,6) selected for validation; these sites are compared to loci 3,4 showing positive h-MACC values, validated earlier (Supplementary Fig. 12). We note that all the sites used in this analysis are H3-enriched, i.e. DNA protection against digestion comes from the same class of proteins (histones). **(c)** H3 tag frequencies (NGS data) and 'raw' qPCR values at the tested sites of positive and negative h-MACC. The qPCR enrichments (y-axis) were computed relative to genomic DNA. Increase and decrease in qPCR signal upon MNase concentration increase correspond to the negative and positive h-MACC respectively. The results show marked difference between the sites of positive and negative h-MACC. **(d)** The scores computed similarly to h-MACC but using qPCR values (qPCR-MACC) differ significantly between negative and positive groups. Thus, qPCR results fit the quantitative expectations from the MACC calculation (see Supplementary Fig. 20 for further details).

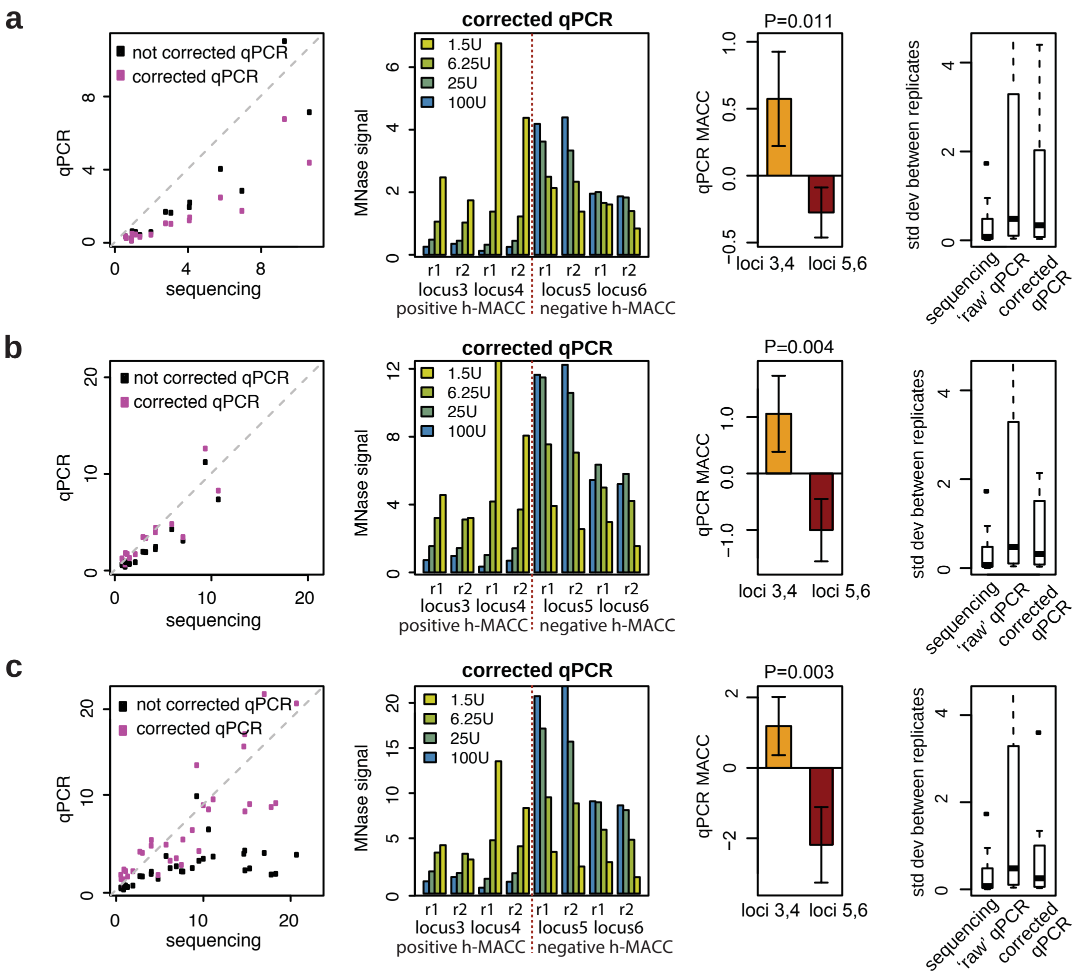

Supplementary Figure 20.

The correction of qPCR results to facilitate cross-platform comparison.

(a-c) Corrected qPCR signal better correlates with the sequencing results (cf. Supplementary Fig. 19c,d); three correction schemes were used (described below). The panels left to right: scatter plot showing correspondence between qPCR and NGS signals; corrected qPCR values for the sites of positive and negative h-MACC (loci 3,4 and loci 5,6 respectively, see Supplementary Figs. 12,19); comparison of the corrected qPCR-MACC for positive and negative h-MACC sites; comparison of the variability in the qPCR signal before and after correction. The used corrections are based on the assumption that the bias in qPCR results would disproportionally increase the signal obtained under light digestion conditions (see Supplementary Fig. 19a). This bias originates from the fact that the amplified sequences can be embedded in longer fragments (and detected by qPCR). The correction algorithms were devised to test this assumption and to determine whether the qPCR data offered robust validation of the NGS data. The correction algorithm used in (a): the patterns of qPCR and sequencing signals are similar at the sites of positive h-MACC (monotonic increase in the signal at loci 3,4). Therefore the correction model was trained on the sites of positive h-MACC and then applied to the sites of negative MACC. We note that the trends observed at the positive h-MACC sites are not distorted substantially (i.e. there is no over-correction). Specifically, for each sample the ratios between the tag frequencies (NGS data) at highest MNase concentration and other digestion conditions were computed at each positive h-MACC site. Then, the correction coefficients required for obtaining the same ratios at these sites for qPCR signal we computed (three correction coefficients per site per sample, corresponding to the MNase concentrations 25U, 6.25U, and 1.5U). Finally the correction coefficients were averaged for each MNase concentration and applied to correct the qPCR signal at the negative h-MACC sites. The correction algorithm used in (b): instead of computing ratios between sequencing tag frequencies as in (a), a linear model fitting qPCR values at the positive h-MACC sites on the NGS tag frequencies at the same set of sites was built for each MNase point. This model was used to correct qPCR signal at both the sites of positive and negative h-MACC. Training the model on the sites of positive h-MACC only allows 'unsupervised' correction at the sites of the negative h-MACC. The correction algorithm used in (c): the same as in (b), but the linear model was trained on both the sites of positive and negative h-MACC together. While this approach is based on the same 'training' and 'test' data sets, it uses maximal number of data points for computing correction coefficients and thus provided the most complete model. All used correction procedures lead to similar results and conclusions, confirming our assumptions and validating the results.

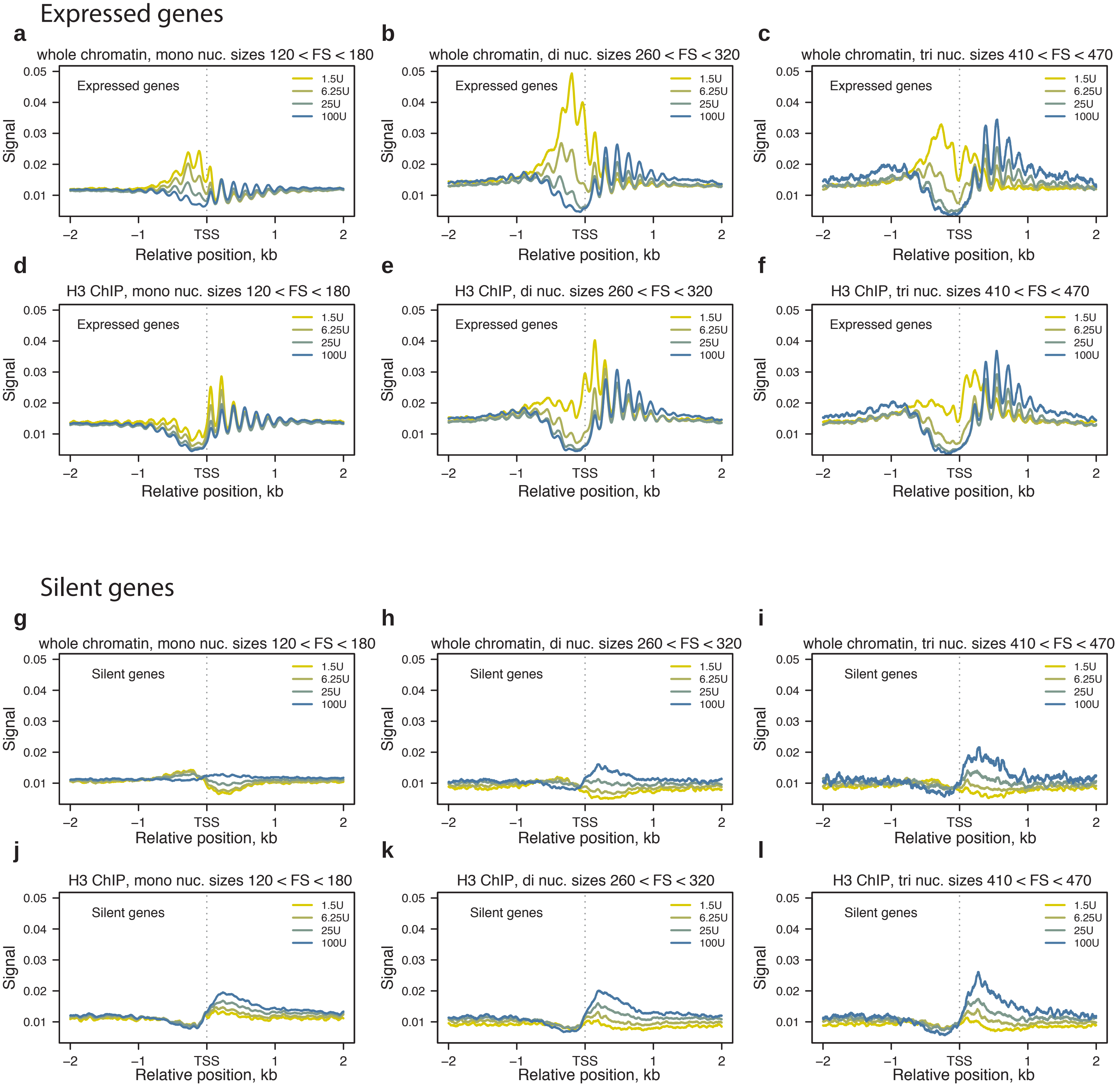

Supplementary Figure 21.

Stratification of the MNase profiles at gene starts by the fragment lengths.

(a,d,g,j) fragments of mono-nucleosomal sizes (120bp < fragment size < 180bp); **(b,e,h,k)** fragments of di-nucleosomal sizes (260bp < fragment size < 320bp); **(c,f,i,l)** fragments of tri-nucleosomal sizes (410bp < fragment size < 470bp). The profiles are shown for expressed (a-f) and silent (g-l) genes. Yellow-blue color scheme indicates MNase concentration levels (1.5, 6.25, 25, 100 U/uL).

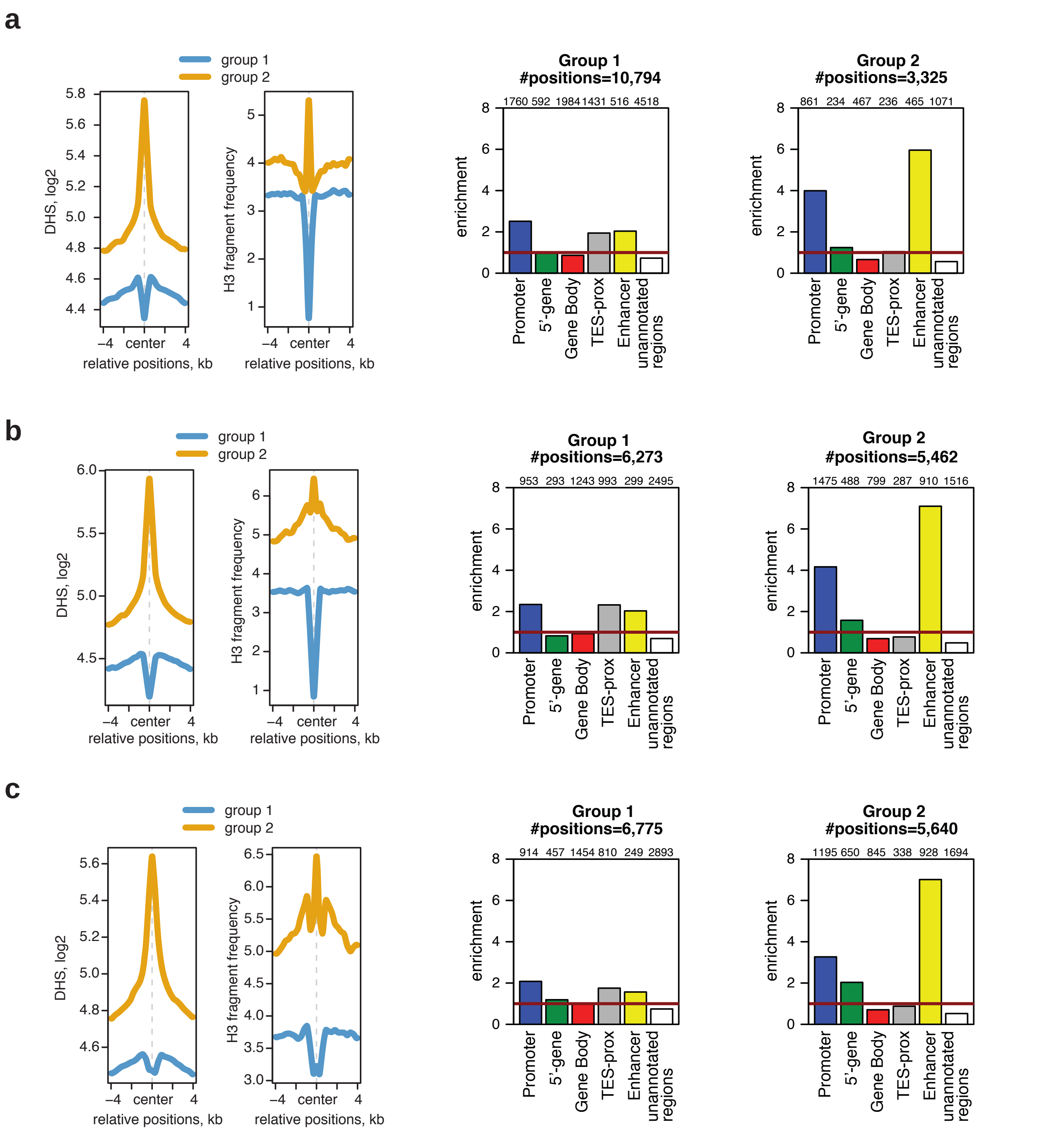

Supplementary Figure 22.

Properties of the sites from group 1 and 2 identified with the data stratified by the digestion fragment size.

(a) Mono-nucleosomes: 120bp < fragment size < 180bp, **(b)** di-nucleosomes: 260bp < fragment size < 320bp, and **(c)** tri-nucleosomes: 410bp < fragment size < 470bp). Left panels represent DNaseI and H3 signals around MACC peaks from group 1 and 2. Right panels represent genomic distributions of the sites from group 1 and group 2. The enrichments were computed relative to the expected values for each type of genomic regions. The horizontal red lines provide reference of 1 (‘no enrichment’).

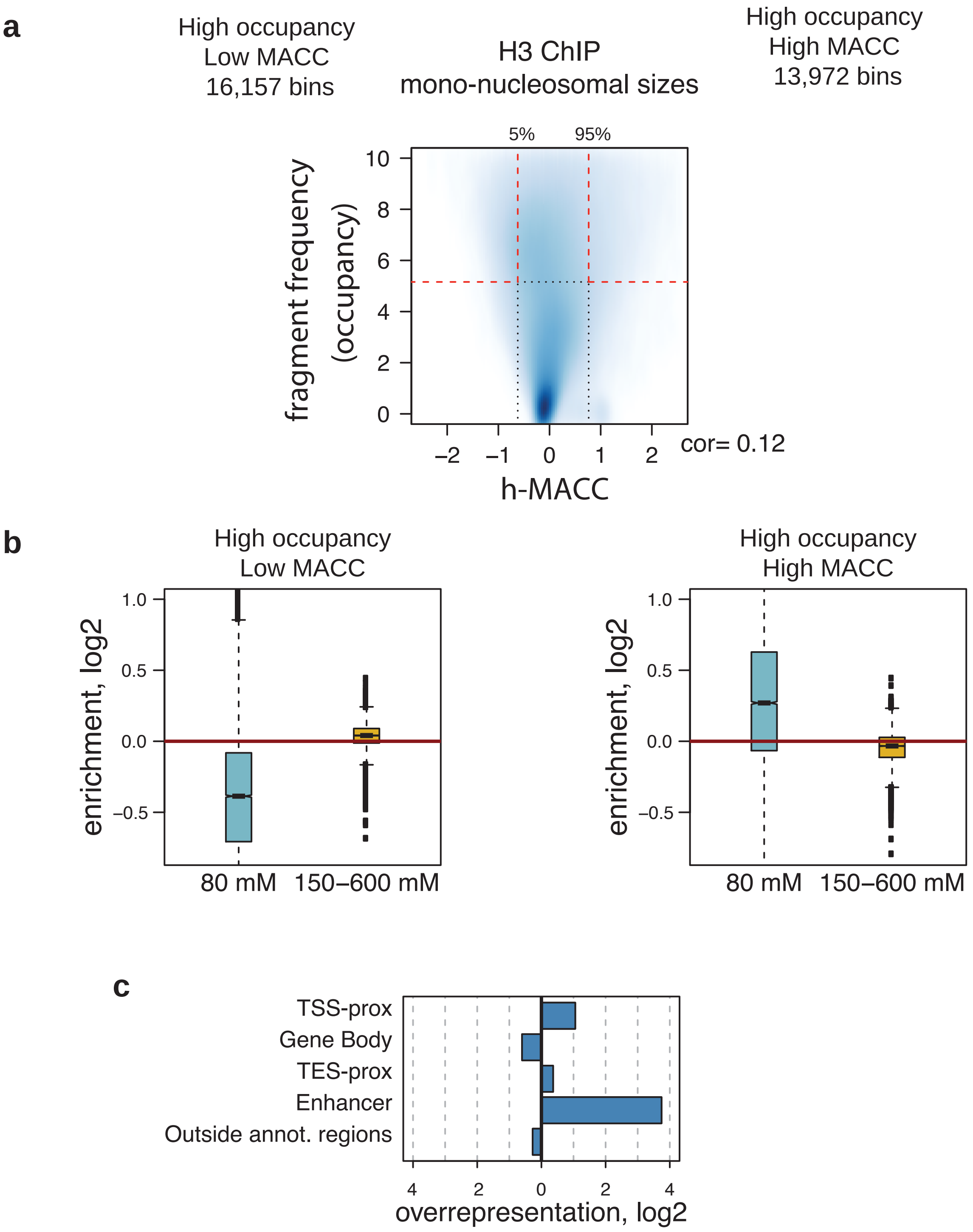

Supplementary Figure 23.
Relation between h-MACC and nucleosome occupancy based on the data set comprising digestion fragments of mono-nucleosomal size (120bp < fragment size < 180bp).
(a) The scatter-plot of h-MACC and nucleosome occupancy estimated in 300-bp bins. The horizontal dashed line indicates the high occupancy cutoff (top 20% of bins), and the vertical dashed lines indicate MACC values thresholds for top nd bottom 5% of bins. The numbers of genomic bins in each region are indicated on the plot. **(b)** Enrichment of salt-extracted chromatin fractions in the high occupancy bins shown in (a). **(c)** Comparison of the enrichment of the two selected sets of bins in annotated genomic regions. See legend to Figure 4 in the main text.

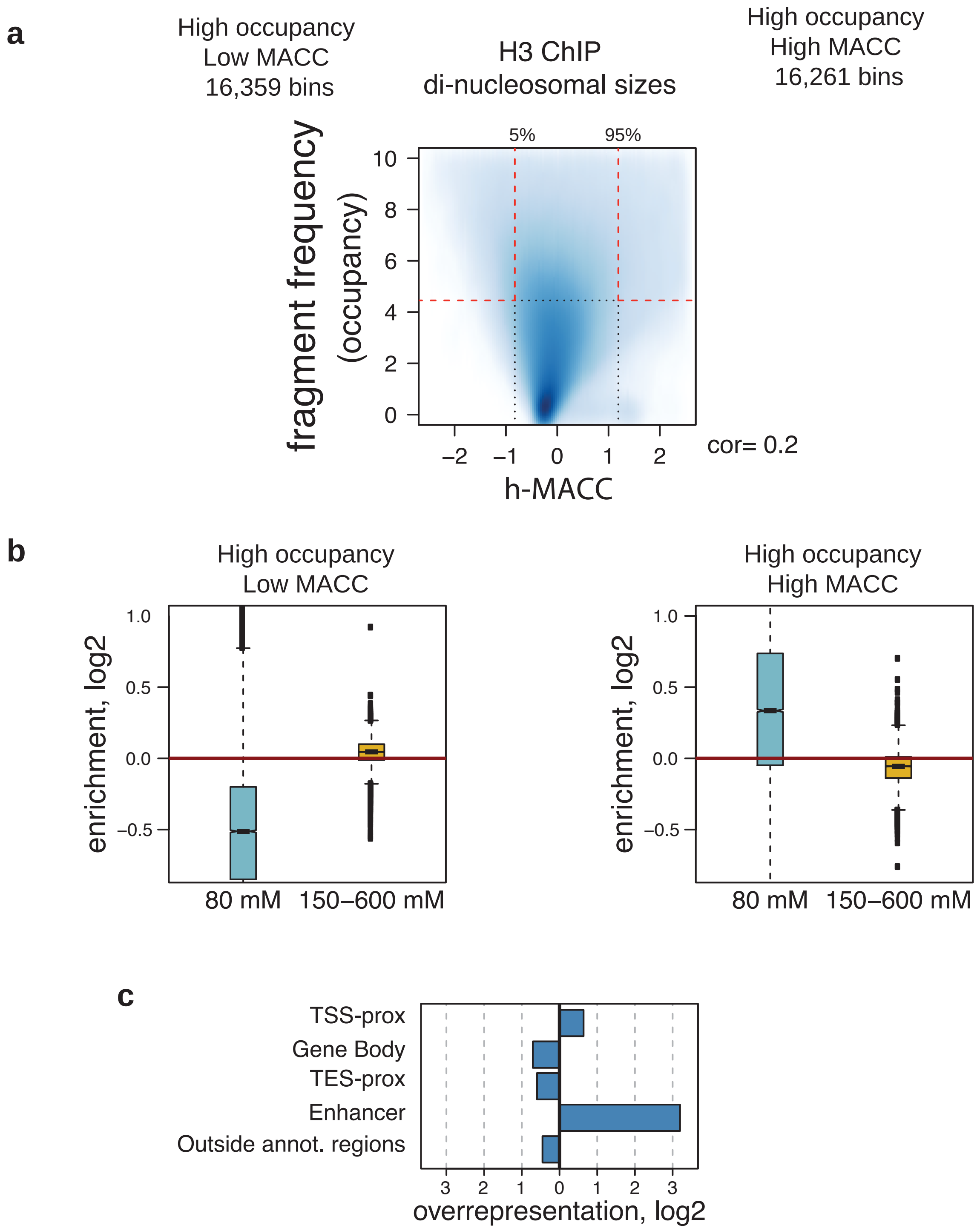

Supplementary Figure 24.
The same as Supplementary Figure 21 for di-nucleosomal fragments (260bp < fragment size < 320bp).

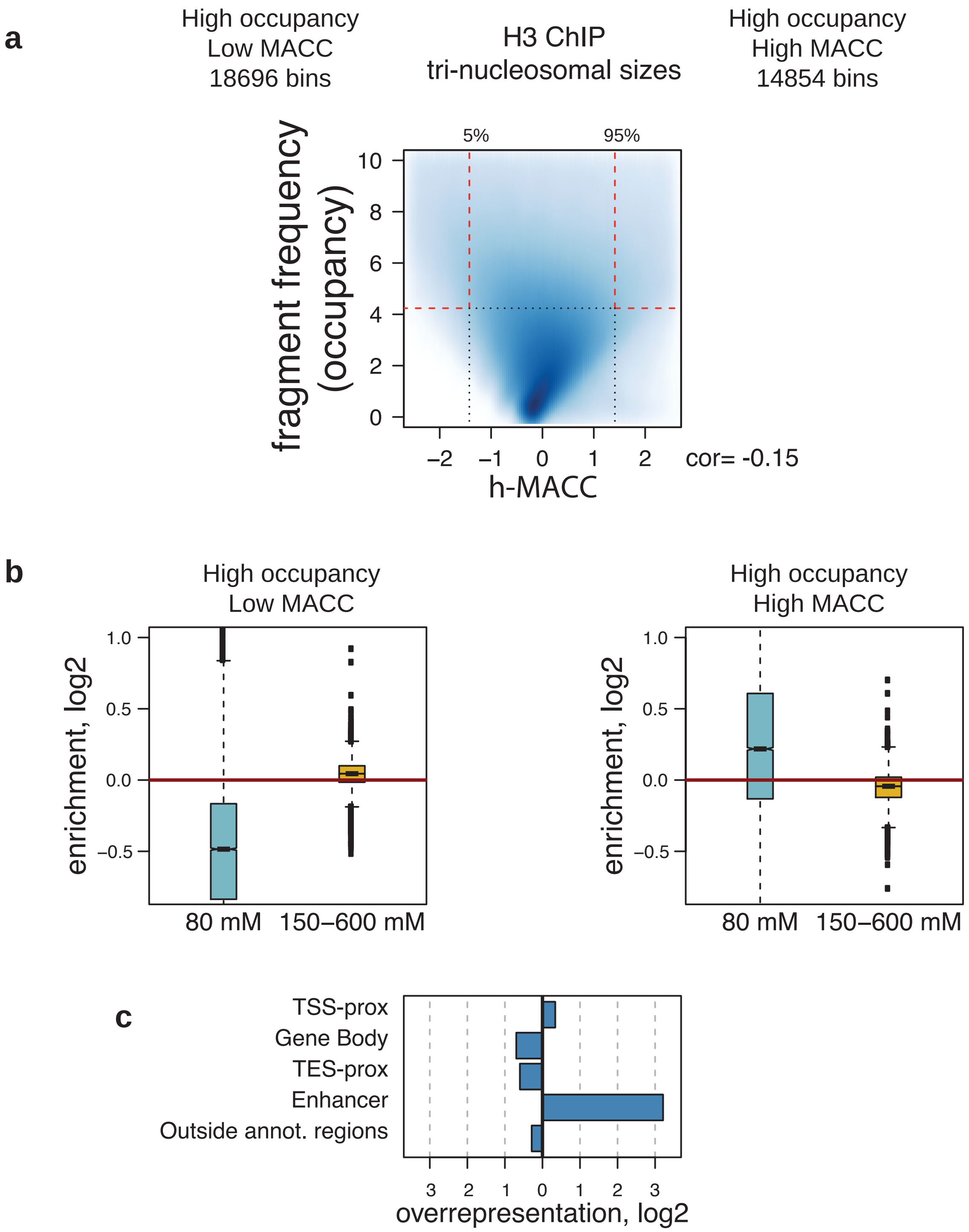

Supplementary Figure 25.
The same as Supplementary Figure 21 for tri-nucleosomal fragments (410bp < fragment size < 470bp).

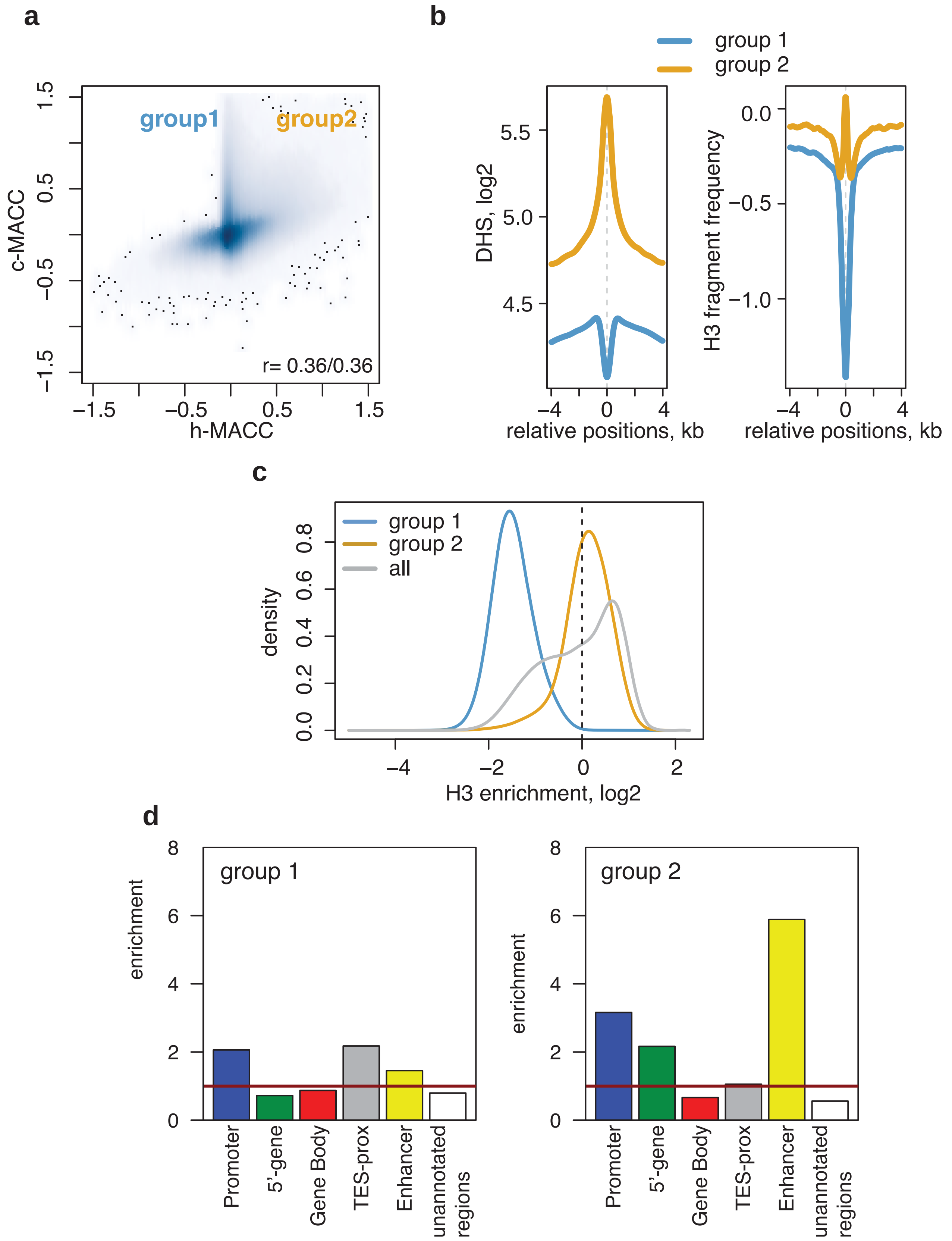

Supplementary Figure 26.
MACC peaks identified at 100bp resolution.
(a) Comparison of c- and h-MACC. **(b)** DHS and H3 signals around group 1 or group 2 peaks. **(c)** H3 enrichment levels. **(d)** Genomic distribution of the MACC peaks from groups 1 and 2. See legend to main-text Figure 3 for more details.

Supplementary Table 1. Counts of aligned sequenced reads in the analyzed data sets.

			**mn 1.5U**	**mn 6.25U**	**mn 25U**	**mn 100U**
s2	whole chromatin (input for H3 and H4 ChIP)	replicate 1	36,888,647	35,094,619	36,680,723	39,521,368
s2	whole chromatin (input for H3 and H4 ChIP)	replicate 2	25,143,991	35,691,263	31,948,788	41,770,198
s2	H3 ChIP	replicate 1	28,371,180	28,114,095	38,155,100	51,383,051
s2	H3 ChIP	replicate 2	34,187,023	34,381,483	41,929,952	40,232,282
s2	H4 ChIP	replicate 1	27,782,531	32,351,775	29,856,567	64,282,019
s2	H4 ChIP	replicate 2	33,313,025	37,508,751	36,063,388	44,435,569
s2	whole chromatin, 'additional' set	replicate 1	46,195,548	41,617,691	45,037,247	47,570,307
s2	whole chromatin, 'additional' set	replicate 2	46,931,099	54,402,611	57,759,996	53,314,128

			**mn 1U**	**mn 4U**	**mn 16U**	**mn 64U**
mouse ESC	whole chromatin	replicate 1	44,578,085	57,573,117	51,448,957	51,568,490
mouse ESC	whole chromatin	replicate 2	43,036,686	45,781,700	63,622,422	51,672,391
mouse ESC	whole chromatin	replicate 3	54,327,028	63,465,684	69,299,812	69,666,391
mouse ESC	whole chromatin	replicate 4	69,745,132	59,252,672	12,688,370	41,927,778
mouse NPC	whole chromatin	replicate 1	48,772,050	54,579,943	52,184,775	51,408,755
mouse NPC	whole chromatin	replicate 2	41,177,960	49,380,654	53,003,017	61,198,788
mouse NPC	whole chromatin	replicate 3	59,235,653	62,342,642	63,230,890	61,291,432
mouse NPC	whole chromatin	replicate 4	58,803,459	50,774,685	27,975,994	45,716,577

			**mn 5.4U**	**mn 20.6U**	**mn 79.2U**	**mn 304U**
K562	whole chromatin		25,294,403	86,491,647	55,047,453	56,653,717

Supplementary Table 2. Camparison of c-MACC profiles computed with different numbers of titration points.

replicate 1

replicate 2	mn1.5U mn6.25U mn25U mn100U	mn1.5U mn6.25U mn25U	mn6.25U mn25U mn100U	mn1.5U mn25U	mn6.25U mn100U	mn1.5U mn100U	mn6.25U mn25U	mn1.5U mn6.25U	mn25U mn100U
mn1.5U mn6.25U mn25U mn100U	0.87	0.82	0.68	0.82	0.68	0.86	0.56	0.71	0.35
mn1.5U mn6.25U mn25U	0.82	0.81	0.58	0.81	0.58	0.81	0.5	0.73	0.26
mn6.25U mn25U mn100U	0.72	0.62	0.67	0.62	0.67	0.71	0.5	0.49	0.39
mn1.5U mn25U	0.82	0.81	0.58	0.81	0.58	0.81	0.5	0.73	0.26
mn6.25U mn100U	0.72	0.62	0.67	0.62	0.67	0.71	0.5	0.49	0.39
mn1.5U mn100U	0.86	0.82	0.67	0.82	0.67	0.86	0.55	0.71	0.34
mn6.25U mn25U	0.62	0.57	0.52	0.57	0.52	0.61	0.43	0.47	0.26
mn1.5U mn6.25U	0.67	0.69	0.41	0.69	0.41	0.67	0.38	0.66	0.16
mn25U mn100U	0.38	0.29	0.43	0.29	0.43	0.37	0.27	0.2	0.3

*c-MACC values were computed independently for each biological replicate based on all four, three, or two MNase titration points. Each c-MACC profile was GC-corrected independently. MNase titration points used to compute each of c-MACC profiles are indicated in the top row (for replicate 1) and the first column (replicate 2). The Pearson's correlation coefficients between the corresponding MACC profiles are shown in each table cell.
